# Identification, molecular characterization and expression of aminopeptidase N-1 (APN-1) from *Anopheles stephensi* in SF9 cell line as a candidate molecule for developing a vaccine that interrupt malaria transmission

**DOI:** 10.1186/s12936-020-03154-3

**Published:** 2020-02-19

**Authors:** Javad Dadgar Pakdel, Sedigheh Zakeri, Abbasali Raz, Navid Dinparast Djadid

**Affiliations:** 1 Malaria and Vector Research Group (MVRG), Biotechnology Research Center (BRC), Pasteur Institute of Iran (PII), Pasteur Avenue, P.O. Box 1316943551, Tehran, Iran; 2 Trauma Research Center, Sina Hospital, Tehran University of Medical Sciences, Hassan Abad Square, Imam Khomeini Avenue, PO BOX: 1136746911, Tehran, Iran

**Keywords:** Malaria, *Anopheles stephensi*, Aminopeptidase-N1, *Spodoptera frugiperda* (Sf9) insect cell line, VIMTs

## Abstract

**Background:**

According to the World Health Organization reports, billions of people around the world are at risk for malaria disease and it is important to consider the preventive strategies for protecting the people that are living in high risk areas. One of the main reasons of disease survival is diversity of vectors and parasites in different malaria regions that have their specific features, behaviour and biology. Therefore, specific regional strategies are necessary for successful control of malaria. One of the tools that needs to be developed for elimination and prevention of reintroduction of malaria is a vaccine that interrupt malaria transmission (VIMTs). VIMT is a broad concept that should be adjusted to the biological characteristics of the disease in each region. One type of VIMT is a vector-based vaccine that affects the sexual stage of *Plasmodium* life cycle. According to recent studies, the aminopeptidase N-1 of *Anopheles gambiae* (AgAPN-1) is as a potent vector-based VIMT with considerable inhibition activity against the sexual stage of Plasmodium parasite.

**Methods:**

Systems for rapid amplification of cDNA ends (3ʹ-RACE) and genome walking methods were used for sequence determination of *apn*-*1* gene from *Anopheles stephensi* and distinct bioinformatics software were used for structural analysis. AsAPN-1 was expressed in *Spodoptera frugiperda* (Sf9) insect cell line using the baculovirus expression system. Recombinant AsAPN-1 was purified under the hybrid condition and its biological activity was assayed.

**Results:**

*Asapn*-*1* gene and its coded protein from *An. stephensi* were characterized for the first time in this study. Subsequently, the structural features and immunological properties of its coded protein were evaluated by in silico approaches. Enzymatic activity of the recombinant AsAPN-1, which was expressed in Sf9 insect cell line, was equal to 6 unit/μl.

**Conclusions:**

Results of this study revealed that AsAPN-1 is very similar to its counterpart in *An. gambiae*. In silico evaluation and fundamental data which are necessary for its evaluation as a VIMT-based vaccine in the next steps were acquired in this study and those could be useful for research groups that study on malaria vaccine for countries that *An. stephensi* is the main malaria vector there.

## Background

Despite the impressive progresses in medical sciences, according to the World Health Organization (WHO) report in 2018, malaria is still as one of the most significant infectious diseases in the world and there were 219 million new cases with increment of about 3.5 million cases over 2017 [1, 2].

The severity of the disease is directly related to the species of *Plasmodium* and the geographical dispersion and proportion of *Anopheles*. There are about 30 species of *Anopheles,* which are responsible for transmission of malaria across the world [3]. One of the most considerable challenge in malaria control is the diversity of the vector and parasite species in different endemic regions which means distinct strategies must be considered for each geographical region [4]. For instance, *Anopheles gambiae* is the main malaria vector in sub-Saharan of Africa, while *Anopheles stephensi* is the most prevalent vector which is spread from the east of Africa (Djibouti and Ethiopia) to the south regions of China [5]. In addition, resistance to insecticides and drugs increases the complexity of malaria treatment and control [6–8]. Moreover, proximity with special endemic malaria regions affects the malaria control progress and, therefore, needs more considerations to achieve the final goal. For example, Iran which is in elimination phase is a neighbour of Afghanistan and Pakistan, which are categorized in the “Control Phase” with high prevalence of malaria. This situation might be established in many parts of the world and may lead to the failure of the malaria elimination goals of the United Nations by 2030 [9]. According to the malEra guidelines, vaccines are one of the main tools for malaria control and with regards to the progress in the elimination programme; specific types of vaccines should be considered for each country. Development of the vaccines that interrupt malaria transmission (VIMTs) was emphasized by the vaccine consultative group for countries that have passed the pre-elimination step and have proceeded to the global goal [10]. VIMTs are divided into various types; one type is the classical transmission-blocking vaccine that blocks the sexual parasite development by targeting the required effector molecules in the vector, including mosquito-based transmission-blocking vaccines with targets such as *Anopheles gambiae* aminopeptidase N-1 (AgAPN-1) [11], *CPBAg1* (Carboxy Peptidase B1) [12, 13], *Trypsin* [14], saglin [15], FREP 1 [16, 17], and SGS1 [18]. APN-1 is a candidate molecule for which the blocking efficacy against *Plasmodium falciparum* in *An. gambiae* has been confirmed [19]. Given the fact that Jacalin prevents ookinete attachment by masking the glycan ligands on the surface of midgut epithelial cells, Dinglasan et al. used affinity chromatography to identify its receptor on the midgut epithelial cells of *An. gambiae*. Some glycoproteins, such as APN-1, are receptors for the lectin-similar structures of ookinete that trigger the attachment of ookinetes to the internal side of the epithelium and are essential for preceding the sexual development of parasite in the mosquito midgut [13, 16, 20].

Therefore, Dinglasan et al. used AgAPN-1 as a TBV candidate and their results showed that it can inhibit the sexual development of *P. falciparum* with 100% efficacy (at ~ 10 µg/ml specific IgG). In addition, it was shown that 100 μg/ml of polyclonal antibody against AgAPN-1 could reduce oocyst formation of *Plasmodium berghei* in the midgut of *An. gambiae*. Furthermore, peptide mapping showed that a 135 amino acids fragment located in the N-terminal of AgAPN-1 is immunogenic even in the absence of adjuvant and has equal blocking efficacy similar to the full length of AgAPN-1 [21].

Since *An. stephensi* is the main malaria vector from the east of Africa to the south regions of China, and due to the considerable efficacy of APN-1 as a VIMT in whole protein and sub-unit formats, the *apn*-*1* gene and its related protein in *An. stephensi* were identified and characterized in the current study to provide the basic and fundamental information necessary for developing an effective and regional mosquito-based VIMT in areas that *An. stephensi* is the major threat for malaria transmission.

## Methods

### Primer design

Identification of the middle part of *apn*-*1* mRNA sequence of *An. stephensi* had been reported in the study of Bokharaei et al. [22]. Thus, general and specific primers were designed for performing the 5ʹ-Genome walking and 3ʹ-RACE, respectively, based on the reported sequence using the GeneRunner software (version 4.0.9.68 beta) (Table 1). Their specificity was evaluated by the nucleotide BLAST server (http://blast.ncbi.nlm.nih.gov/Blast.cgi) [13, 23]. After determining the full-length sequence of *apn*-*1* mRNA molecule, specific primers were designed to amplify the full length coding sequence of the mRNA and DNA of the target gene for evaluating the arrangement of introns and exons. In addition, full-length cloning primers, which contained restriction sites, were designed for cloning and expressing of the whole APN-1 in SF9 cell line using the baculovirus expression system.

**Table 1:** List of the primers designed and used in this study

	No.	Primer name	Sequence
5ʹ-Genome walking primers	1	GWA	5ʹ-GATCAGGCGTCGCGTACCTCNNCTACTG-3ʹʹ
	2	GWB	5ʹ-GATCAGGCGTCGCGTACCTCNNCTACT-3ʹ
	3	GWC	5ʹ-GATCAGGCGTCGCGTACCTCNNCTAC-3ʹ
	4	GWD	5ʹ-GATCAGGCGTCGCGTACCTCNNCACGCA-3ʹ
	5	GWE	5ʹ-GATCAGGCGTCGCGTACCTCNNCACGC-3ʹ
	6	GWF	5ʹ-GATCAGGCGTCGCGTACCTCNNCACG-3ʹ
	7	GWG	5ʹ-GATCAGGCGTCGCGTACCTCNNGAGAC-3ʹ
	8	UAP-N1	5ʹ-CTGTGAGCAGTCGTATCCACCGATCAGGCGTCGCGTACCTC-3ʹ
	9	UAP-N2	5ʹ-CCTGTGAGCAGTCGTATCCAC-3ʹ
	10	GW 27-R	5ʹ-ACGGTGAACGTTGCCTTCAG-3ʹ
	11	GW 40 –R	5ʹ-GTTTTCCCAGTCACGAC-3ʹ
	12	GW 59-R	5ʹ-CGATATGGCACTGTAGGTAATGCTG-3ʹ
	13	GW 130-R	5ʹ-GGCGTCTTGTCGAACACCG-3ʹ
	14	GW 282-R	5ʹ-CTCGTCCAGCACCTTCAG-3ʹ
3ʹ-RACE primers	15	RC 921	5ʹ-CAGATCATGCGCACCTGGACCAACG-3ʹ
	16	RC 1011	5ʹ-CTGTTGGCGTTCGTTGTGTC-3ʹ
	17	RC 1200	5ʹ-CGAATTCTATGCCGCCCATCT-3ʹ
	18	RC 1422	5ʹ-TCCTACTTCAACAGCCGTCTCC-3ʹ
	19	RC 2196	5ʹ-CACAAGTACCTCGTGCAGAC-3ʹ
	20	RC 2241	5ʹ-TGGGCTACAAGGACTGTCTG-3ʹ
	21	RC 2328	5ʹ-CCACCGTCACGTACTGTTAC-3ʹ
	22	RC 3146	5ʹ-CATCCCGAACACCACAACCG-3ʹ
	23	Linker Oligo(dT)	5ʹ-GAGATTTGAATCTTGCTTCTGGGCCCTCTAT TGTCATTGTCTTTTTTTTTTTTTTTTT-3ʹ
	24	Inner RC	5ʹ-GGCCCTCTATTGTCATTGTC-3ʹ
	25	Outer RC	5ʹ-GAGATTTGAATCTTGCTTCTG-3ʹ
	26	T7 RC	5ʹ-TAATACGACTCACTATAG-3ʹ
Full-length primers	27	FL inner-F	5ʹ-CGATTAGTGCACCACCGAGTCTGC-3ʹ
	28	FL Outer-F	5ʹ-ATGGTGCGTTCGAAGATCCTAGCAG-3ʹ
	29	FL inner-R	5ʹ-CCTAATCATCTCGATTCCGGAGCTCCTG-3ʹ
	30	FL outer-R	5ʹ-TTATCCCAGTAGATGGACCGCG-3ʹ
Full-length cloning primers	33	UFLC-F	5ʹ-AAGCTTctATGGTGCGTTCGAAGATC-3ʹ
	34	DFLC-R	5ʹ-CTCGAGtTTATCCCAGTAGATGGACCG-3ʹ

### Collection of *Anopheles stephensi* samples and insectary rearing

All experiments were performed on the *An. stephensi mysorensis,* using the Chabahar strain which had previously been collected from the Chabahar district in the southeastern part of Iran, Sistan and Baluchestan Province. This strain was reared in the National Insectarium at Pasteur Institute of Iran (PII), Malaria and Vector research Group (MVRG) under the standard conditions: a temperature range of 26–28 °C, 60–80% humidity, and 12 h light/dark cycle [13]. Five-days adult mosquitoes were used in the all experiments of this study.

### RNA and DNA extraction

Live mosquitoes were anesthetized on ice, and their midguts were dissected. Ten isolated midguts were used for RNA extraction which was performed by High Pure RNA Tissue Kit (Roche, Germany) according to the manufacturer’s instruction. The extracted RNAs were treated with DNase I enzyme to remove any probable DNA contamination, as per the instruction recommended by manufacturer (Thermo Scientific, USA). Quantity and quality of the extracted RNA was evaluated using the Colibri microvolume spectrometer (Titertek-Berthold, Germany) and 1.5% agarose gel electrophoresis, respectively. Genomic DNA, which was used to determine the full length of *apn*-*1* gene sequence and arrangement of its introns and exons, was extracted from the whole body of the mosquitoes by MBST genomic DNA extraction kit (MBST, Iran).

### Reverse transcription (RT; cDNA synthesis)

Considering that there was no data on the expression pattern of *apn*-*1* gene, total RNA was extracted in different time frames (0, 2, 7, 12, and 18 h) after blood feeding. RT reaction was performed in a final volume of 20 µl using the Oligo(dT) as primer. The volume of 200 ng of the total RNA was adjusted to 5 µl by adding RNase-free distilled water. This mixture was incubated at 75 °C for 5 min to remove the secondary structures and was cooled on ice immediately. Then, RT mix which included Revertaid Moloney murine leukaemia virus [M-MuLV], RNase inhibitor, deoxynucleoside triphosphate solution, RT buffer, and Oligo(dT) primer, was added to the cooled RNA, and RT reaction was started by the following program: 10 min at 25 °C, 60 min at 42 °C, and 10 min at 70 °C. All reagents were purchased from Takara, Japan.

### Polymerase chain reaction (PCR) assay

All reactions were carried out in a 20 μl total volume for 35 cycles. Ingredients of the PCR reactions consisted of 400 nM of each primer, 1 unit of Taq DNA polymerase, 0.2 mM of each deoxynucleoside triphosphate, 2.5 µl of 10× reaction buffer, 1.5 mM MgCl_2_, and 150 ng of genomic DNA in each reaction as template. The PCR reactions were performed in a Flex Cycler PCR machine (Analytik Jena, Germany) using 5 min primary denaturation at 94 °C which followed by 35 cycles of denaturation at 94 °C for 30 s, annealing at 58–62 °C for 40 s depending on the Tm of primers and 1-3 min (with regard to the size of amplicon) extension at 72 °C with an additional final extension at 72 °C for 10 min. Finally, PCR products were evaluated using the 1.5% agarose gel.

### 3ʹ-RACE and 5ʹ-genome walking

Based on the study of Raz et al. [13], the RT reaction was performed by the linker primer (Table 1). Various gene specific primers were designed for this technique. In this step, two PCR reactions were carried out with outer and inner primers as reverse and internal gene-specific primers as forward primers. After performing the agarose gel electrophoresis, only amplicons that their sizes were close to the expected size were selected for TA cloning. Selected amplicons were recovered from agarose gel and TA cloned in pTG19-T vector (both from Vivantis, Malaysia). Next, TA-cloned products were sequenced by Macrogen company (South Korea). After editing sequences using Chromas software (Technelysium, Australia), those were analysed by nucleotide BLAST (http://blast.ncbi.nlm.nih.gov/Blast.cgi) for similarity search.

To determine the 5ʹ-end sequence of the *apn*-*1* gene, the genome walking method was used according to Alipour et al. [23]. Briefly, the prepared cDNA with linker Oligo(dT) primer was used as template. Asymmetric PCR was fulfilled with the universal (UAP-N1for step one and UAP-N2 for step two) and gene-specific primers (GW 27-R GW 40-R, GW 59-R, GW 130-R, and GW 282-R) in two consecutive steps. Then, amplicons with the sizes more than the expected size were selected, TA-cloned and sequenced. Finally, sequences were analysed by Gene Studio Software 2.1.1.5 (GeneStudio Inc., USA), and those had an overlapped region with the middle part sequence of the *apn*-*1* mRNA molecule were selected for further analysis.

### Assembly and determination of the full-length sequence of *Asapn*-*1* mRNA molecule

After sequencing of the 3ʹ- and 5ʹ-ends of As*apn*-*1* mRNA molecule, acquired sequences were assembled using the GeneStudio software. Finally, FL Inner-F/FL Inner-R and FL Outer-F/FL Outer-R primers were designed for amplifying and sequencing the full-length sequence of the mRNA and DNA of *Asapn*-*1* gene.

### In silico study

#### Analysis of the sequence features

First, to determine the structural features of the AsAPN-1 protein and its division, the coding sequence of *Asapn*-*1* mRNA was translated to its coded residues by GeneRunner software 4.0.9.68 Beta. Then, the protein BLAST was used to search similar proteins to our query. Subjects with high similarity and scores were selected to perform multiple alignments with Clustal Omega based on the Clustal W method and determine the conserved residues. Evaluation of the antigenicity and finding the functional epitopes of the target molecule was accomplished by Hopp-Woods-Hydropathy plot as a subdivision of ExPASy protscale tool (http://web.expasy.org/protscale/) [24]. In the output plot, a negative value is associated with non-polar residues, values >0 represent the hydrophilic ß strands regions and negative values indicate the ɑ helix hydrophobic regions [25]. In addition, the physiochemical properties such as amino acid quantity, instability index and estimated half-life were analysed using the stand alone Protparam (https://web.expasy.org/protparam/).

#### Prediction of the post translation modifications (PTMs)

Post-translational modifications (PTMs) of proteins are usually accomplished by proteolytic cleavage or covalent modifications. These modifications are done by specific enzymes that identify especial amino acid residues or distinct sequences. In order to predict the amino acids that undergo glycosylation, NetNGlyc 4.0 and NetOGlyc 3.1 servers were used [26]. The NetOGlyc server is based on the neural network predictions of mucin type GalNAc O-glycosylation sites. In this method, the score of each amino acid is calculated, and values above the threshold are reported as glycosylated amino acids.

#### Prediction of the dynamic nature of AsAPN-1

The residue-level backbone dynamics from the AsAPN-1 sequence in the form of backbone N–H interaction was predicted, to understand the motion restriction of an atomic bond in comparison with the molecular reference frame [27]. The values were variable between 1, for fully restricted (rigid conformation), and 0, for fully random movement (highly dynamic). DynaMine webserver (Brussels-, Belgium) was used to assess the dynamic nature of AsAPN-1.

#### Peptide toxicity prediction

Support Vector Machines (SVM) classifier was used from the freely available software package SVM^light^ for searching the toxic motifs in our target protein by the MEME Suite software (version 3.5.0), and then query sequences were hit with the toxic peptide motif list using the MAST software [28]. If hit was higher than the SVM score threshold, which is 5, peptides are predicted toxic for the host cell.

#### Prediction of the three-dimensional (3D) structure of AsAPN-1

The SWISS-MODEL (http://swissmodel.expasy.org) was used for predicting the 3D structure of our target protein [29]. Prediction of the 3D structure was performed based on the X-ray crystallography deposited data of the well-characterized molecules using this method [30]. According to the QMEAN (qualitative model energy analysis) and GMQE (global model quality estimation) scores, the best model was selected and used for comparing the structural properties of the AsAPN-1 with the reference molecule in the next steps [31]. UCSF Chimera software version 1.11.2 was used for alpha carbon root-mean-square deviation (Cɑ-RMSD) and overall RMSD analysis [32].

#### Molecular docking studies

In order to evaluate the structural predictions and determining the structurally important residues in the active site of AsAPN-1, we docked the active sites of AsAPN-1 and AgAPN-1 with bestatin, which is the aminopeptidase enzymes specific inhibitor with the EADock method using the SwissDock server [33]. First, the crystal structure of bestatin was retrieved from the 2DQM accession number (PDB database). Then, the entire protein surface was scanned using the SwissDock to identify the most probable interaction sites with bestatin. This scanning is performed based on multi-objective hybrid evolutionary algorithm. In this method, the probability of binding the ligand to receptor in the form of random and semi-random is examined at two levels of accuracy. In the first stage, screening is faster and has less accuracy (simple-Fitness), but in the second stage, screening is time-consuming and has high-precision (full-Fitness). The best appropriate ligand binding site is calculated based on the highest free energy which determined by the analytical Generalized Born Molecular Volume performed in the CHARMM program among the different binding probabilities. This sampling and selecting of the target cavity is performed with the accuracy of 2 angstroms in the crystal structure [34].

For AsAPN-1, the mapped protein onto the 3D grid and ligand connection cavities were evaluated and the amount of binding powers were finally determined as a free energy of amino acid geometries and specific ligand interaction positions.

#### Prediction of antigenic peptides of AsAPN-1

With regard to the importance of developing the subunit vaccines, the profile of antigenic peptide of full-AsAPN-1 was evaluated using the Immunomedicine group tool (http://imed.med.ucm.es/Tools/antigenic.pl) which determines the antigenic peptides according to the Kolaskar and Tongaonkar method with 75% accuracy [35]. In addition, this analysis was performed for AgAPN-1 as the reference molecule and performing further comparison.

#### Prediction of the epitope avidity

After introducing the antigens in the antigen presenting cells, antigen processing and presenting are done in this type of immune cells on the major histocompatibility complexes (MHCs). Two factors are important in antigen presenting which are the primary structure of the peptide and allelic variation of the residues in the binding site of the major histocompatibility complex receptor. These two elements should interact together for final presentation of the small processed peptides. Therefore, we evaluated the potential presentation of our target protein with the common MHC-II alleles in Iran using the NetMHCII 2.2 and pyDockWEB web based servers to evaluate the induction of potential humoral response. To perform this evaluation, the MHC-II binding regions of AsAPN-1 and their binding affinities with receptors were predicted in the first step. According to the recommendations of Vina et al. [36], the prevalent alleles of HLA in Iran were selected. Subsequently, the binding affinity between the peptide segments and prevalent alleles were investigated using the NetMHCII 2.2 and pyDockWEB web based servers. NetMHCII 2.2 predicts the binding efficacy of peptides to HLA-DR, HLA-DQ, HLA-DP alleles using the artificial neuron networks. The prediction values are given in nanomolar IC50 values, and binding efficiency are determined by the strong and weak values [37]. pyDockWEB, as a structure-based server, was used for predicting the MHC-II binding properties with an overall ΔG which includes electrostatics, desolvation energy, and limited van der Waals contribution [38]. In addition, these analysis and evaluations were performed for AgAPN-1.

#### Baculovirus expression and purification

For expression of the recombinant AsAPN-1, UFLC-F, and DFLC-R primers were used (Table 1). Expand High Fidelity Taq DNA polymerase (Roche, Germany) was used to amplify the coding sequence of *asapn*-*1* gene. Then, this amplicon was TA cloned in pTG-19 vector and subcloned to pFastBack HT A vector using SpeI and XhoI restriction enzymes (Invitrogen, Germany). Next, pFastBac-full apn construct, was first transformed to *Escherichia coli* DH5α and the purified pFastBac-full apn was transformed to *E. coli* DH10Bac subsequently and confirmed using sequencing. The generation of recombinant virus and transposition was performed according to the recommendations of Invitrogen for Bac-to-Bac system (http://www.ThermoFisher.com). The screening and presence of the recombinant bacmid (pFastBac-full apn) was done by colony-PCR using M13 Forward and Reverse primers. Then, recombinant bacmid was purified from one of the selected colonies and transfected into the Sf9 cells using the Cellfectin II (Invitrogen). This step was performed in order to generate the recombinant baculovirus stock P1. P1 viruses were harvested and stored at 4 °C. In order to earn the highest yield of recombinant virus, the optimal multiplicity of infection (MOI) was determined by screening the 1,3,5,7 MOIs. To determine the expression kinetics of the target recombinant protein, screening was preformed within 0 until 96 h of post infection using SDS-PAGE. Afterwards, P1 stock virus was used to get the P2 stock in the fresh Sf9 cells at 27 °C for maximum 96 h and similarly P2 virus was used for producing the high titer of P3. Finally, P3 viruses were harvested and used for recombinant protein expression according to the Invitrogen protocol (http://www.ThermoFisher.com). For recombinant AsAPN-1 expression analysis, 1 ml of the harvested culture was analysed using the SDS-PAGE analysis. Solubility of the expressed recombinant protein was determined according to the QIAexpressionist protocols (http://kirschner.med.harvard.edu/files/protocols/QIAGEN_QIAexpressionist_EN.pdf) [39]. With regard that 6X His-tag was embedded in the upstream of the target protein by the used expression system, Ni-NTA (QIAGEN, Germany) beads were used for protein purification under the hybrid purification condition according to the Life Technologies™’ instruction with few modifications in buffer ingredients [40]. Hybrid purification method is a type of purification that the 3D structure and native folding of the target protein is rearranged beyond denaturation and no need to renaturing after purification. Different buffer ingredients were: lysis and denaturing binding buffer contained 100 mM NaH_2_PO_4_, 10 mM Tris·Cl, 8 M urea and 5–8 mM imidazole at pH 8.5; denaturing wash buffer contained 100 mM NaH_2_PO_4_, 10 mM Tris·Cl, 8 M urea at pH to 6.3; and native elution buffer contained 50 mM NaH_2_PO_4_, 300 mM NaCl, 250 mM imidazole at pH 8.5. In brief, cultured cells were collected from the 75 cm^2^ cell culture flasks which contained 2 × 10^6^ cells/mL and pelleted cells were lysed using 6 ml of lysis and denaturing binding Buffer. Then, lysis solution was centrifuged at 8000 rpm and 4 °C for 15 min. Next, the supernatant was incubated at 23 °C for 60 min with Ni-NTA agarose beads while those were rotating. After that, Ni-NTA agarose beads were washed three times with denaturing wash buffer. In the last step, target protein was eluted using the native elution buffer. The total purified protein was desalted using the Econo-Pac^®^ (Bio Rad, USA) and finally analysed using SDS-PAGE. To keep the bioactivity of the purified AsAPN-1 for long term and storage, our target protein was mixed with glycerol (5–50% (w/v)) and divided in small aliquots and kept in − 20 °C until use.

#### Analysis of bioactivity

For analysis of the bioactivity of AsAPN-1, a spectrophotometry based test which l-leucine p-nitroanilide substrate (Sigma) is broken down by the endopeptidase property of the aminopeptidase enzyme was used. This endopeptidase activity of the enzyme causes an increment of absorbance at 405 nm wavelength due to the release of 4-nitroaniline [41]. In order to determine the bioactivity of recombinant AsAPN-1, 1 μg/ml of the purified protein was used and *Streptomyces griseus* aminopeptidase (Sigma) and 1,10-phenanthroline (10 μM; Sigma) were used as positive control and metalloprotease inhibitor, respectively. Eighty microliters of the purified recAsAPN-1 which contained 1 μg/ml of active enzyme, 20 µl of 10 mM l-leucine-p-nitroanilide as substrate (Sigma, USA) and 100 µl of 50 mM Tris-HCl buffer (pH 7.6) were used to perform the bioactivity assay. This mixture was incubated at 37 °C. AsAPN-1 activity was measured spectrophotometrically using the microtitre plate (BioTek Instruments, USA) and reading the absorbance at 405 nm in 15, 30, 90 and 120 min after the start of reaction. This reaction was also performed in the presence of 10 μM 1,10-phenanthroline as an inhibitor simultaneously.

## Results

### Characterization the 3ʹ-and 5ʹ-end sequences of *asapn*-*1* mRNA molecule

After confirmation with the internal primers, the amplicon of RC-921 and inner primers with ≈ 900-bp length was sequenced and nucleotide BLAST analysis revealed its high similarity with its counterpart in *An. gambiae*. Finally, this sequence was submitted to the GenBank under the 1990583 accession number.

After performing the genome walking, amplicons with length greater than 500-bp (six amplicons), were sequenced. Nucleotide BLAST revealed that all of the acquired sequences were related to *Asapn*-*1* gene. Therefore, final contig from the assembled amplicons was submitted to the GenBank under the 1985952 accession number.

### Characterization of the full-length of *asapn*-*1* mRNA sequence

After sequence determination of the 3ʹ- and 5ʹ-ends of the *asapn*-*1* mRNA molecule, these sequences were assembled, and the full-length sequence of *asapn*-*1* mRNA was determined and submitted to the GenBank (accession number: 2017959). The length of this sequence was 3285-bp which included an open reading frame with 3078-bp length that encoded a protein with 1025 residues, and predicted molecular Pi (isoelectric point) and molecular weight equal to 4.82 and 118,792.27 Da (118.79 KDa), respectively. Comparison of the coding sequences of *asapn*-*1* on DNA and mRNA sequences revealed that the *asapn*-*1* gene has four introns and five exons and introns have been located at 698-794, 1241-1477, 1822-1891 and 3218-3250 positions (GenBank accession number: 2017959). Sequence analysis of *apn*-*1* gene introns demonstrated that their donor and acceptor splice sites were AG-AC and CA-TG, GT-AG, and AG-TC, respectively. Comparison of the splice sites of *asapn*-*1*, *agapn*-*1* (XM_318000.4), and similar gene in *Anopheles culicifacies* (MK033514.1) revealed that the sequences of donor and acceptor of the splice sites in *An. gambiae* and *An. culicifacies* are more similar (Table 2).

**Table 2:** Comparison of three *Anopheles* species donor and acceptor splice sites

Anopheles species	Splice sites 1	Splice sites 2	Splice sites 3	Splice sites 4
*An. stephensi*	AG-AC	CA-TG	GT-AG	AG-TC
*An. gambiae*	GT-AG	TA-GG	GT-AG	GT-AG
*An. culicifacies*	*GT*-*AG*	GT-AG	GT-AG	GT-AG

**Fig. 1:**
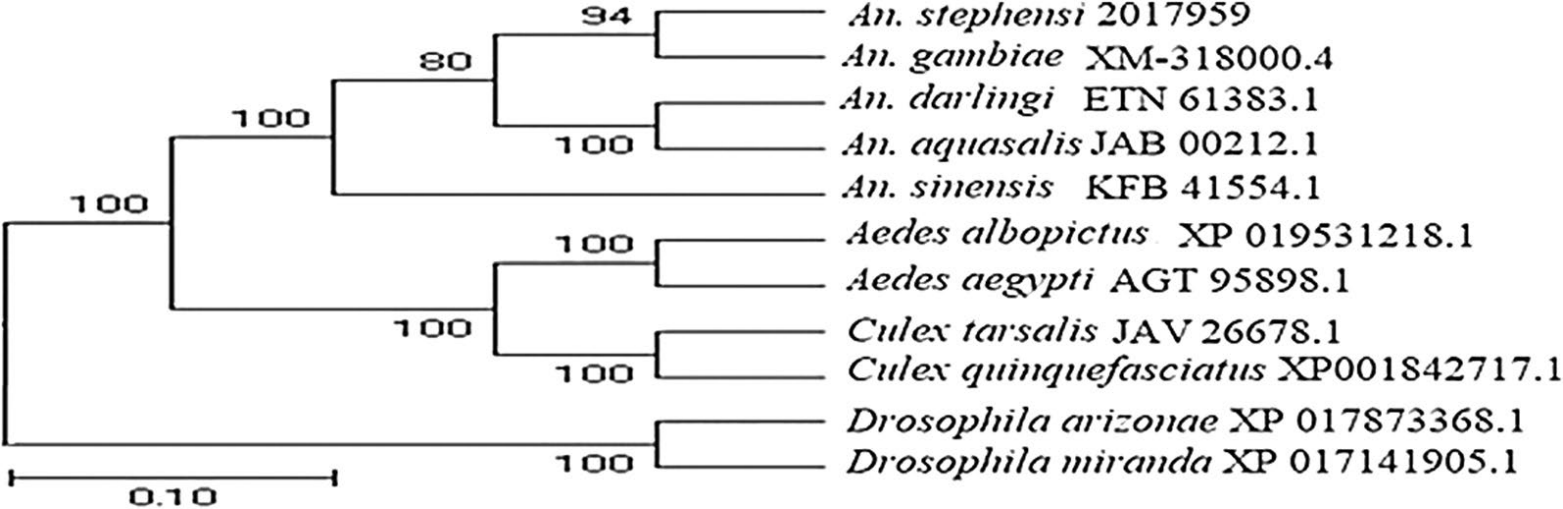
Phylogenetic tree analysis of the characterized *apn*-*1* genes from different insects. Characterized *apn*-*1* genes from different insects were aligned and compared using the MEGA7.0 software based on the neighbour-joining method with 1000 bootstrap replicates. The accession number of each sequence is presented in the front of the insect name. The repeat percentages of branches in which the associated taxa clustered together are shown next to each branch. The scale bar is equal to 0.1 changes per nucleotide

Phylogenetic tree analysis of the nucleotide sequences of our target gene and other *apn*-*1* genes from the other insects indicates that the *asapn*-*1* is located in *Anopheles* group and has close relationship with *An. gambiae* (Fig. 1).

### In silico findings and predictions

**Fig. 2:**
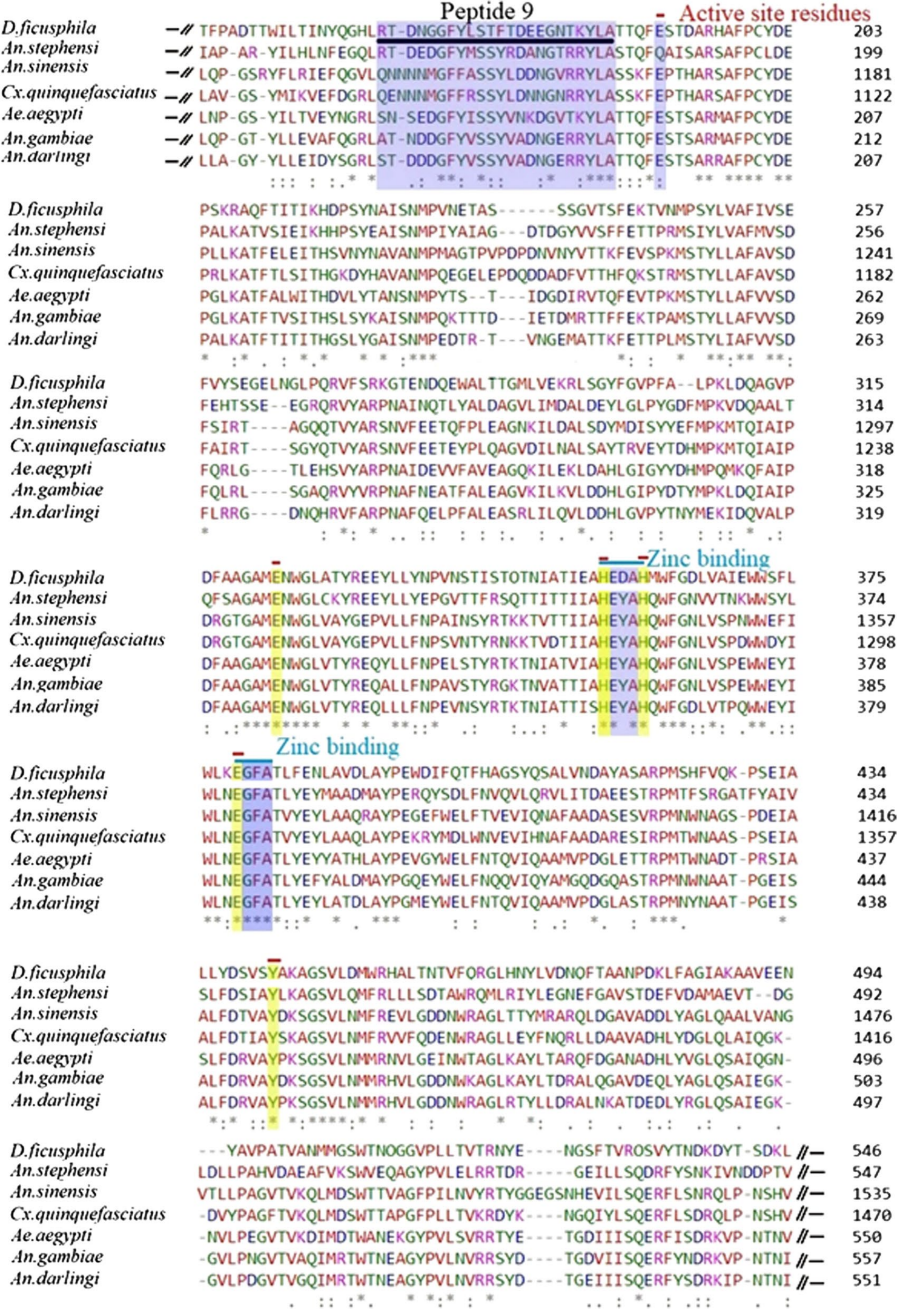
Alignment of the AsAPN-1 with seven structurally similar proteins. Alignment was carried out using the Clustal Omega based on Clustal W multiple sequence alignment method. Conserved residues are highlighted in yellow, and the degree of similarity is shown by different signs (asterisk, colon, and dot) from high to low, respectively. Involved residues in zinc-binding domain, active site, and catalytic domain are indicated by blue, red, and black lines, respectively, peptide 9 is highlighted by a purple rectangle box

Alignment of *asapn*-*1* mRNA sequence with *agapn*-*1* mRNA sequence revealed that their similarity is 80.48%. The comparison of AsAPN-1 with AgAPN-1 (XP_318000.4), and APN-1 of *An. culicifacies* (QCO76330.1) showed that these proteins have 74.93% and 73.31% similarity with AsAPN-1, respectively. Alignment of AsAPN-1 with seven similar proteins revealed that the structurally important motifs of these proteins, such as zinc binding domains, active site and peptide 9 have a high degree of conservation (Fig. 2).

Homology modelling analysis of the target protein with fully characterized proteins in structure through the SWISS-MODEL server revealed that the highest structural similarity is related to homo-dimer of APN-1 from the *An. gambiae* (AgAPN-1) (PDB accession No: 4WZ9). Therefore, the AgAPN-1 was selected as the reference molecule to predict the structural features of AsAPN-1.

### Analysis of the predicted 3D structure of AsAPN-1

**Fig. 3:**
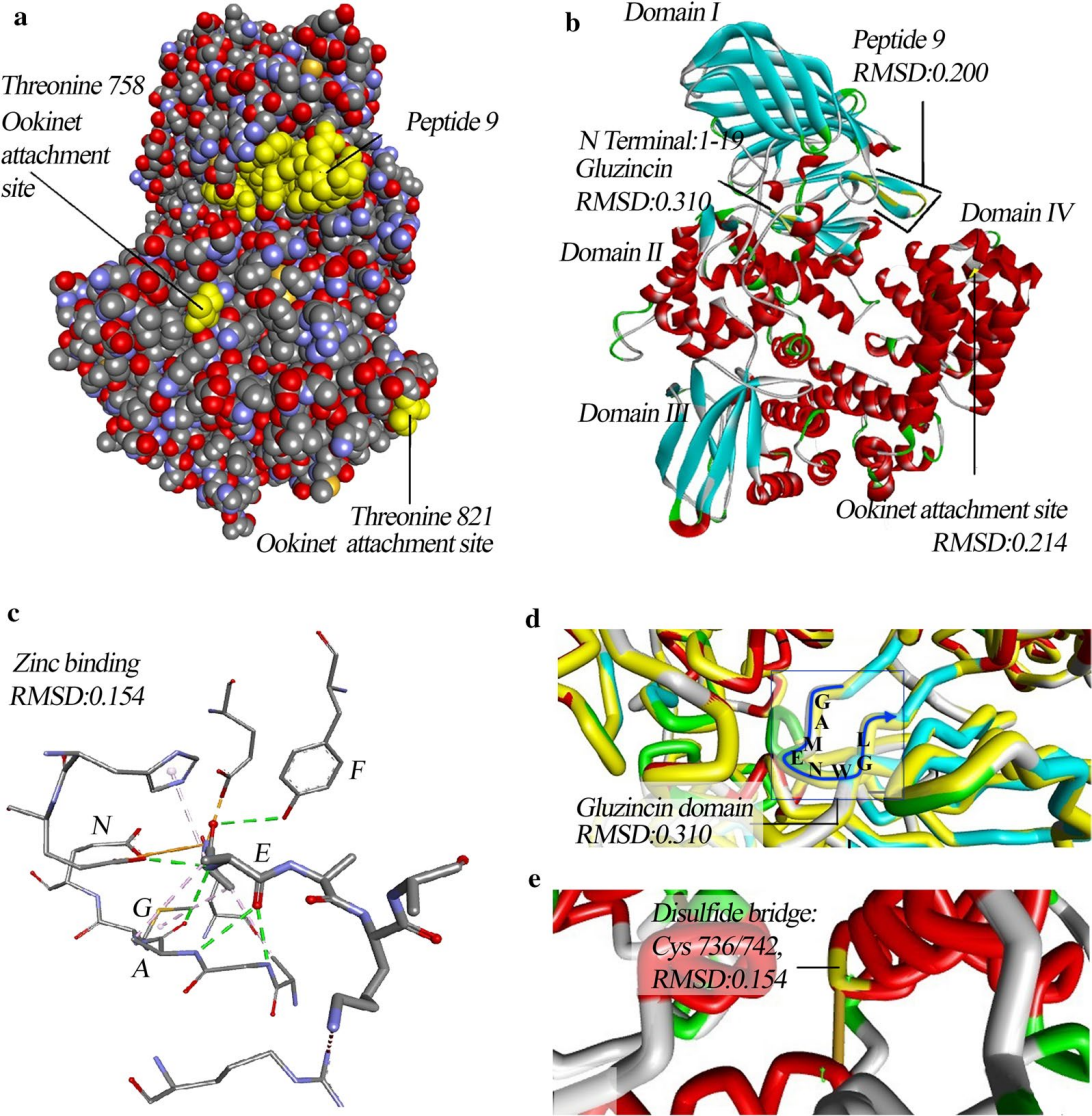
Topology structures and superimposition of AsAPN-1 and AgAPN-1. **a** AgAPN-1 (4WZ9 PDB) structure as reference molecule; **b** close-up view of superimposition of the ookinete attachment site; **c** close-up view of superimposition of the zinc-binding site **d** close-up view of superimposition of Gluzincin which is conserved domain in M1 metalloproteases; **e** close-up view of the superimposition of cysteine which participate in disulfide bridge construction

**Fig. 4:**
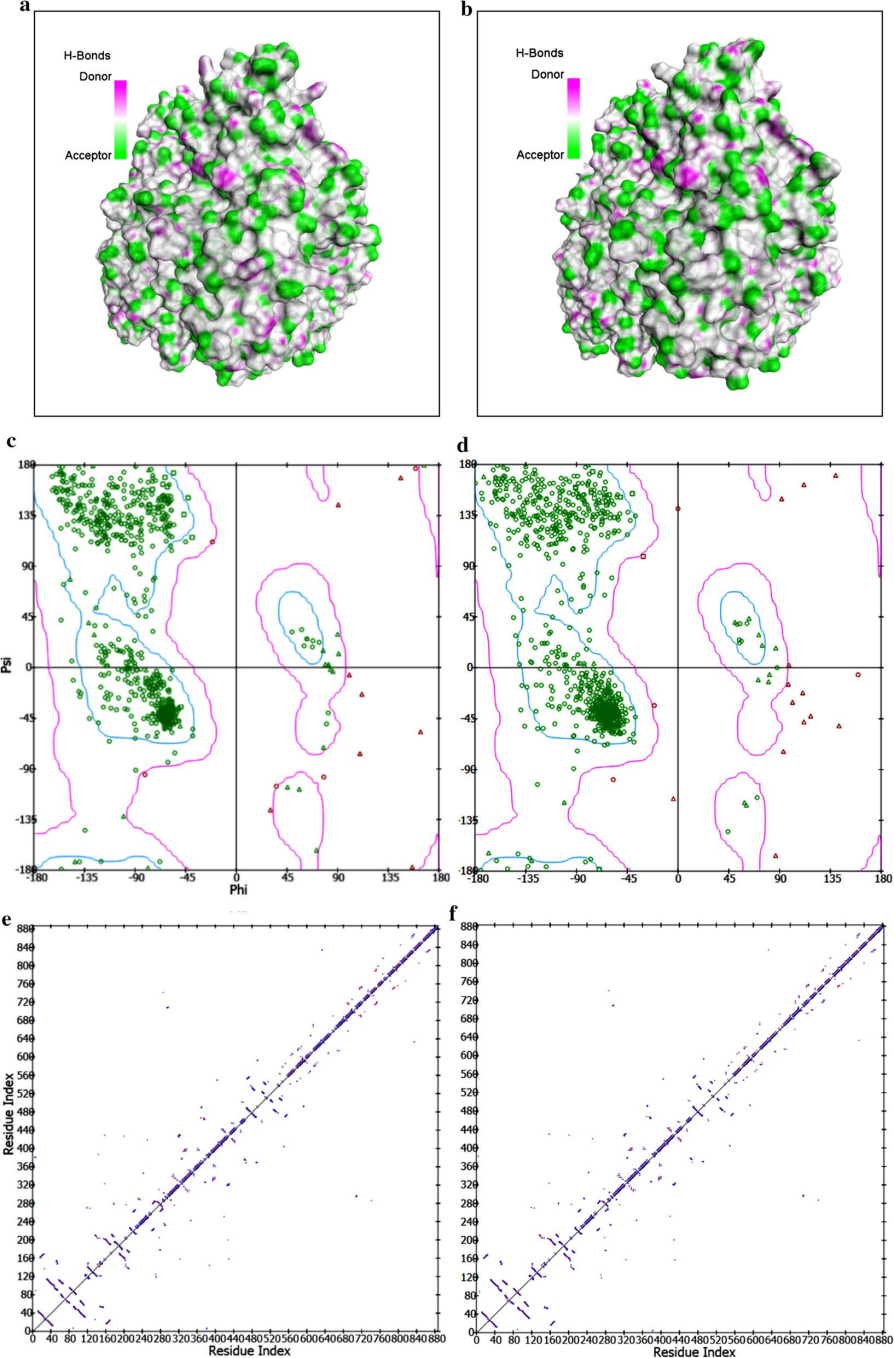
Structural comparison of the AsAPN-1 and AgAPN-1 (as reference molecule). Hydrogen bonds donor and acceptor plot of AsAPN-1 (**a**) and AgAPN-1 (**b**) show that the involved residues in creating the hydrogen bonds are very similar in both molecules; Ramachandran plots: the Ramachandran plots of AsAPN-1 and AgAPN-1 were analysed by discovery studio visualizer and revealed that the pattern of AsAPN-1 (our target molecule) (**c**) is very similar to the AgAPN-1 as the reference molecule (**d**). Green spots which are enclosed in blue lines, indicate that residues are in the most favored region, while the spots which are located out of the redlines are in the disallowed regions. Stoichiometry analysis of Carbon-α: the stoichiometry analysis of alpha carbons in AsAPN-1 (**e**) and AgAPN-1 (**f**) revealed that the topologies of carbons-α in both molecules are very similar. All these results were obtained using the discovery studio visualization software (Version 4.0 Accelrys, San Diego, USA)

**Fig. 5:**
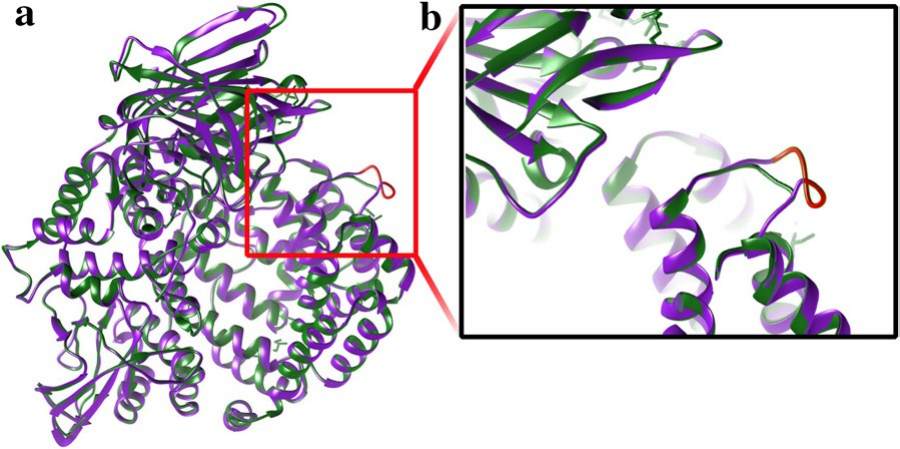
Structural superimposition. **a** Structural superimposition of AsAPN-1 (purple) and AgAPN-1 (green) as a reference molecule revealed that the structural topology of these proteins is very similar together. **b** In addition, superimposition revealed that there are five residues (G_832_TGVE_836_) that there is no counterpart for them in AgAPN-1 and those are seen as a protrusion in AsAPN-1(red region). These residues act as a linker between the two adjacent alpha helixes

Prediction of the 3D structure of AsAPN-1 using the SWISS-MODEL and according to the homology modelling and QMEAN and GMQE scores of the predicted models, revealed that the target protein has a close topology to the AgAPN-1 (4WZ9 PDB accession No). Therefore, 4WZ9 was selected as the reference molecule for further structural analysis such as superimposition and finding the counterparts of structurally important residues and motifs, such as ookinete attachment sites, peptide 9, Gluzincin, active site and zinc binding domain (Fig. 3a–e). Structural comparison of AsAPN-1 and AgAPN-1, revealed that our target protein is composed of an N-terminal (residues 1-19), a C-terminal ecto-domain (residues 36-1005) and a glycosylphosphatidylinositol-anchor (residues 1006-1031) (Fig. 3b). As APN-1 has consisted of two symmetric units (A and B homologues) (Fig. 3a, b). Ectodomain (residues 69-954) is composed of four domains (I–IV), which have metallopeptidase M1-family activity. Domain I (residues 69-281) is predicted that composed of a 15 β-sheet (Fig. 3b). Domain II is the catalytic domain (residues 282-534) which contains the substrate recognition site (H377EYAH381) and zinc-binding motif (NE400GFA). Domain III and domain IV (the C-terminal region) encompass the residues 535-625 and residues 626-954, respectively. It is predicted that the active site is located in domain II, and zinc ion is coordinated with the NEGFA motif and the N-terminus of the modeled protein (Fig. 3c). RMSD of the structurally critical residues of AsAPN-1 and AgAPN-1 are presented in Table 3. Moreover, analysis of the reactive residues in hydrogen binding plot (Fig. 4a, b), the torsional angles (Ramachandran plot) (Fig. 4c, d) and stoichiometry analysis of the alpha Carbons of AsAPN-1 and AgAPN-1(Fig. 4e, f) revealed that those have the same patterns. Further analysis revealed that AsAPN-1 has five residues (G_832_TGVE_836_) more than AgAPN-1 in domain II which acts as a linker between the two alpha-helixes and superimposition reveled that it had no effect on the three-dimensional structure of AsAPN-1 and these five residues are seen as a protrusion in the protein structure in comparison to AgAPN-1 (Fig. 5).

**Table 3:** RMSD values of the structurally important residues between the AsAPN1 and AgAPN1 proteins

No.	Position on the AgAPN1	Sequence/amino acid	Position on the AsAPN1	RMSD (mean)	Sequence importance
1	330–337	GAMENWGL	336–343	0.310	Gluzucin
2	366–389	HEY_2_AH_17_E	372–395	0.154	Zinc binding
3	758	T	764	0.126	Ookinet.attachment
4	821	T	827	0.214	Ookinet.attachment
5	736	Cys	742	0.115	Disulfide.bridg.
6	743	Cys	749	0.085	Disulfide.bridg.
7	772	Cys	778	0.308	Disul.bridg.
8	808	Cys	814	0.179	Disul.bridg.
9	179–193	YVSSYVADNGERRYL	185–199	0.200	Immunogen

### Molecular docking analysis

Docking analysis of AgAPN-1 and AsAPN-1 as receptors with bestatin as ligand (specific inhibitor) showed that bestatin interacts with our target and reference proteins in a similar pattern and with the same residues in their active site cavities. The details of these interactions such as estimated ΔG and involved residues are given in Table 4.

**Table 4:** Docking analysis of bestatin as specific inhibitor with AgAPN-1 and AsAPN-1 as its receptors

Anopheles species	Involved amino acids in interaction with bestatin	Diagram of the 2D interactions in active site cavities	Average full fitness (Kcal/mol)	Estimated Δ*G* (Kcal/mol)
*An. gambiae*	Glu 199 Glu 389 His 366 His 370 Glu 333 Tyr 452		− 3239.46	− 8.37
*An. stephensi*	Glu 205 Glu 394 His 371 His 375 Glu 338 Tyr 457		− 3239.40	− 8.30

### APN-1 surface features

**Fig. 6:**
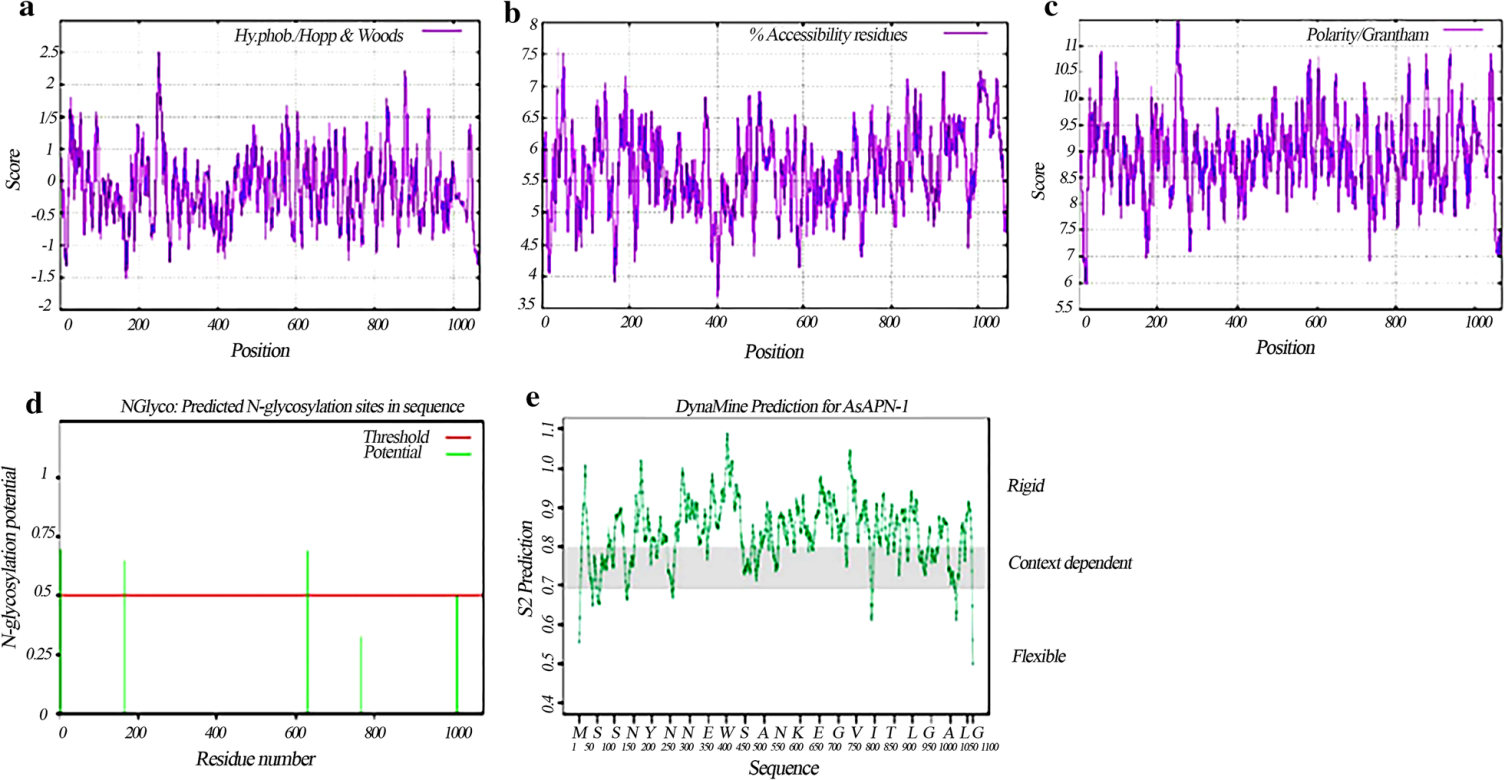
Features of the secondary structure of AsAPN-1 (**a**) Hopp and Woods (1981) hydrophobicity plot of AsAPN-1. A negative value is associated with the non-polar residues and hydrophobic regions and values greater than 0 represent the hydrophilic regions. All residues which are located in the N-terminal and C-terminal of the AsAPN-1 have suitable hydrophilicity. **b** Accessibility of AsAPN-1 analysis using the Protscale-ExPASy server revealed that the most of residues which are located in the N-terminal and C-terminal of AsAPN-1 are accessible in the secondary structure and have a good position for presentation to immune system. In this prediction, values greater than 0 have suitable accessibility for immune system. **c** Amino acid polarity was determined by Protscale-ExPASy. Our results showed that the 230 and 200 residues of the N-terminal and C-terminal of AsAPN-1 are polar, respectively. **d***N*-glycosylation; this analysis was performed by NetNglyc 1.0 server. Our analysis revealed that V2, N148, and N589 positions have acceptable score for N-glycosylation. **e** Prediction of dynamic nature of AsAPN1 using DynaMine server: Our results showed that the most of regions of AsAPN-1 are rigid, and there are only four flexible regions with the lowest predicted S2 value which are M1(0.59)-V2(0.61), G763-(0.62), Y764(0.62) and A975 (0.61)-G976(0.61) residues. In addition, there are two more flexible residues with the lowest S2 value which are L1024 (0.56)-G1025(0.50)

After identifying the AsAPN-1 and matching with AgAPN-1 as reference protein, its structural features such as hydrophobic regions, accessibility, amino acid polarity, and *N*-glycosylation were determined (Fig. 6a–e). Hydrophobicity and hydrophilicity of AsAPN-1 revealed that the most of hydrophilic residues are located at the N-terminal (such as peptide 9) and C-terminal regions. Therefore, they have the numeric values above the zero and AsAPN-1 is considered as hydrophilic antigen (Fig. 6a). In addition, accessibility prediction shows that second structure of AsAPN-1 in all part of N/C terminal would be accessible for the immune system (Fig. 6b). Polarity prediction tools showed that 230 primary residues, residues from 450 to 750 and 800 to 1000 are polar and in terms of electric charge, those are suitable for interaction with immune system (Fig. 6c).

Prediction of *N*-&*O*-glycosylation sites revealed that three amino acids in AsAPN-1 structure are susceptible for *N*-glycosylation that are V2, N148 and N589 (Fig. 6d). Stability of the candidate molecule is one of the basic principles for vaccine development and our prediction shows that in overall, As APN-1 has high stability with eight exceptions that are in the form of four paired flexible regions. These regions are M1-V2, G763-Y764, A975-G976 and finally L1024-G1025 residues (Fig. 6e).

### Prediction of antigenic peptides of AsAPN-1

Prediction of the antigenic peptides of AsAPN-1 and AgAPN-1 using the Kolaskar and Tongaonkar’s method revealed that the profile of their antigenic peptides are very similar together and those have been located in the same topological position in the first structure of two proteins (Additional files 1, 2). AsAPN-1 average antigenic propensity is 0.9956 and 25 immunogenic epitopes were identified. Some of these peptides were short and some of them were long (Table 5) [42]. In comparison with AgAPN-1, it was observed that AsAPN-1 had more and stronger immunogenic components with lower energy (Table 6, Additional files 3, 4).

**Table 5:** Antigenic plot of AsAPN-1

Protein	N	Start position	Sequence	End position
AsAPN-1	1	4	SKAVTVGCCAA	14
	2	35	GSVGVAVNA	43
	3	45	RKTSVHYD	52
	4	60	SGSVAVTT	67
	5	70	HTRGVSS	76
	6	83	VTGAVGD	89
	7	114	GYVSSYVA	121
	8	134	ARMACYD	140
	9	142	KATTVSTHST	151
	10	176	SSYAVVSD	183
	11	188	TRVYVRN	194
	12	197	TAAGVKKVDHGY	208
	13	227	TYRDAVST	234
	14	242	TTAHYAHW	249
	15	260	ATYYAAHAYAYWN	272
	16	293	SADRVAYKS	301
	17	324	RAGAVDY	330
	18	337	GVNGVTV	343
	19	378	MYNYVHA	384
	20	397	HYRSHACRSTRKHVGYYRV	415
	21	474	NYHDVDAYGTDVVSN	488
	22	490	SHKYVTTSWACSGYKD	505
	23	515	GTGAVHDATVTYCY	528
	24	576	RRRVVAVYSGSRVGVDAYMDAVVNVSS	602
	25	699	TGSAAVT	705

**Table 6:** Prediction of AsAPN-1 and AgAPN-1 presented peptides by common MHC-II alleles in Iran using NetMHCII

AgAPN-1				
MHC Alleles	MHC DP Allele	MHC DQ Allele	MHC DR Allele	Number of peptides
Binding situation	High binders 0 Weak binders 10	High binders 0 Weak binders 35	High binders 94 Weak binders 226	687
Highest affinity (nM)	207.9	54.7	4.3	
AsAPN-1				
MHC alleles	MHC DP Allele	MHC DQ Allele	MHC DR Allele	Number of peptides
Binding situation	High binders 58 Weak binders 166	High binders 19 Weak binders 145	High binders 290 Weak binders 423	1011
Highest affinity (nM)	5.1	20.5	4.2	

### Prediction of AsAPN-1 epitopes and MHC-II interactions

Prediction of interactions of the AsAPN-1 and AgAPN-1 epitopes with MHC-II using pyDockWEB revealed that similar antigenic peptides and epitopes of both proteins interact with the common MHC-II alleles in Iran. The binding efficiency of interactions is presented by ΔG by the pyDockWEB and details of the best interaction are presented in Table 7. In addition, similar analysis by different method, which was performed using the NetMHCII, showed that the AsAPN-1 and AgAPN-1 have the similar processing and presentation capability by common MHC-II alleles in Iran (Table 6). It is very interesting that the number of presented peptides and high binder peptides are more for AsAPN-1 in comparison with AgAPN-1. Furthermore, the highest affinities among the evaluated peptides for different MHC-II alleles; which is presented by nano molar (nM); are related to the antigenic peptides of AsAPN-1.

**Table 7:** Prediction of interactions of the AsAPN-1 and AgAPN-1 epitopes with MHC-II using pyDockWEB

An.species	Ranking	MHC DR B binding residues	ΔG	MHC DQA binding residues	ΔG	MHC DQB binding residues	ΔG
*Stephensi*	1	E579, D474, A478	− 34.713	T827, G834, Q293	− 30.475	R278, Y277, L276	− 38.209
	2	G529, Q424, V428	− 32.335	Y77, T784, N244	− 28.591	L229, S228, V227	− 36.053
	3	A629, R574, A528	− 32.028	L829, L884, T344	− 28.579	A328, I327, Q326	− 35.164
	4	W679, R574, A528	− 31.593	W933, V934, E394	− 27.632	F378, W377, Q376	− 34.910
*Gambiae*	1	E574, D469, A473	− 43.215	T821, I826, N288	− 42.264	R273, L272, Q271	− 40.466
	2	G524, Q419, G423	− 37.861	Y771, A778, K238	− 41.554	I223, S222, V221	− 33.475
	3	A623, K569, A523	− 34.736	L823, L878, T339	− 39.920	A323, I322, Q321	− 31.378
	4	W673, K569, A523	− 34.131	W937, V928, E389	− 39.837	F373, W372, Q371	− 28.125

### Expression of the full length of AsAPN-1 using the baculovirus expression system

**Fig. 7:**
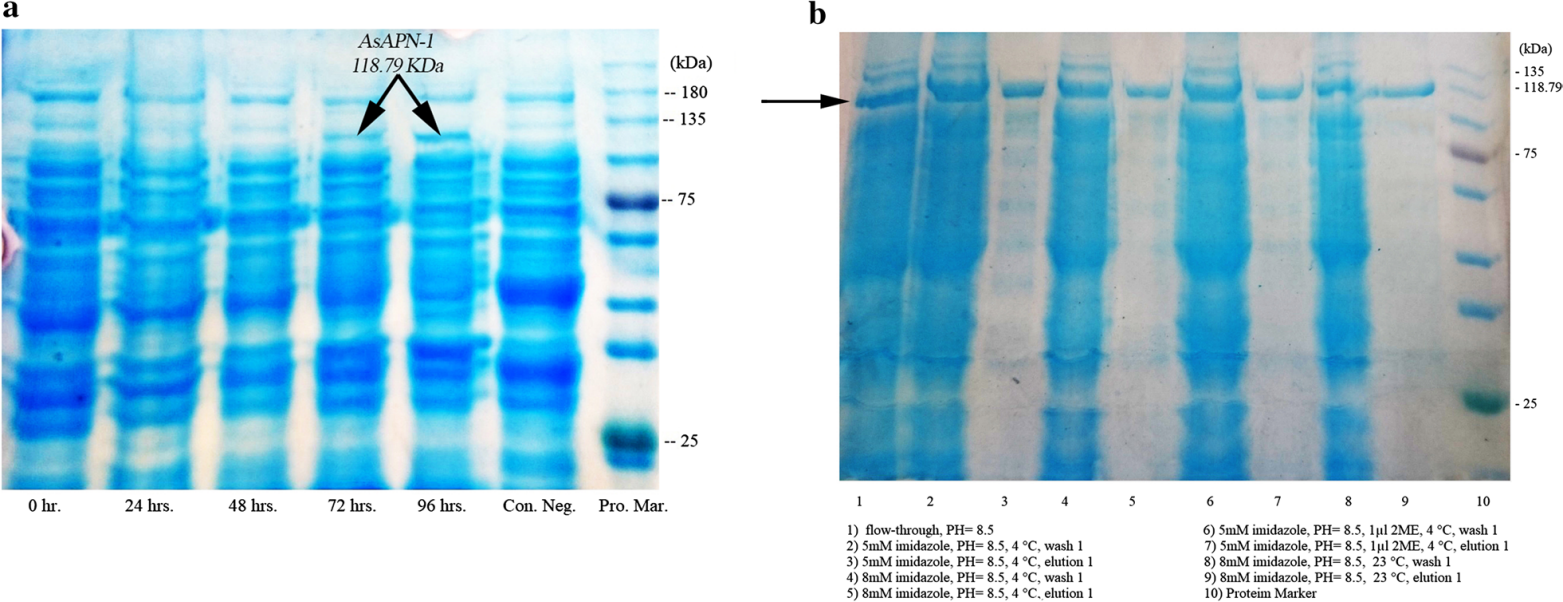
Expression and purification of the full length of AsAPN-1. **a** SDS-PAGE analysis revealed that the recombinant AsAPN-1 has 118.79 kD molecular weight and **b** PH = 8.5 was the most suitable for purification

SDS-PAGE analysis revealed that the recombinant His-tagged AsAPN-1 was produced as non-soluble intracellular molecules in SF9 cell line. The majority of recombinant AsAPN-1 was expressed in the form of inclusion bodies. Therefore, hybrid condition was selected for extraction of AsAPN-1 recombinant protein. The highest protein content was obtained in 96 h after P3 virus inoculation. The best MOI (number of applied viruses per cell) was three, and the best concentration of the heat-inactivated complement fetal bovine serum was 12%. The best temperature and pH for growth and infection of the cultured insect cells in this study were 27 °C and 6.3, respectively. The AsAPN-1 recombinant protein with 118.79 kD molecular weight was produced from the coding sequence of *Asapn*-*1* gene in the SF9 cell line (Fig. 7a).

### Purification of the recombinant AsAPN-1

One of the most important troubles in production of the recombinant AsAPN-1 was disinclination of protein for attachment to Ni-NTA column during purification process. Changing the temperature, increasing the incubation time with Ni-NTA beads, addition of 2-mercaptoethanol, and decreasing the concentration of imidazole did not solve the problem. Finally, this problem was solved by elevating the pH during incubation time with Ni-NTA beads to 8.5 (Fig. 7b).

### Bioactivity assay of AsAPN-1

**Fig. 8:**
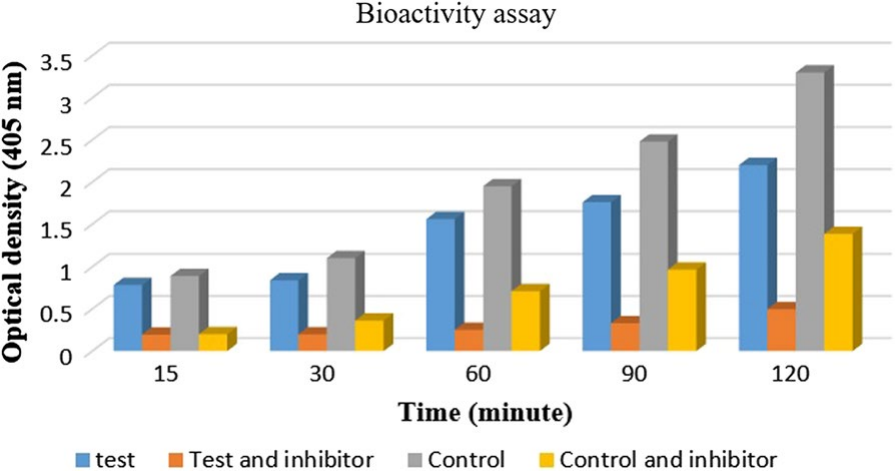
AsAPN-1 bioactivity assay. Bioactivity assay of AsAPN-1 was performed in the presence of the 1,10-phenanthroline as the specific inhibitor, l-leucine p-nitroanilide as specific substrate and *Streptomyces griseus* aminopeptidase (Sigma) as positive control and standard. Bioactivity assay revealed that the recombinant AsAPN-1 could degrade the specific substrate and 1,10-phenanthroline (10 μM) as the metalloproteases inhibitor is able to bind specifically to the active site of recombinant AsAPN-1and disrupt its enzymatic activity. Optical density was measured at 405 nm after 15, 30, 60, 90 and 120 min after the start of reaction

Bioactivity assay revealed that AsAPN-1 and positive control (*Streptomyces griseus*) had the same enzymatic activity in reaction with the specific substrate (l-leucine p-nitroanilide) and inhibitor (1,10-phenanthroline) of aminopeptidase enzymes. Enzymatic activity assay revealed that the amount of bioactivity of AsAPN-1 in comparison with the standard protein is 6 unit/μl. The details of bioactivity assay of AsAPN-1 and *Streptomyces griseus* in the presence of l-leucine p-nitroanilide and 1,10-phenanthroline is presented in (Fig. 8). Bioactivity assay showed that two proteins act on l-leucine p-nitroanilide as specific substrate and 1,10-phenanthroline inhibit their bioactivity on the substrate.

## Discussion

Complexity of *Plasmodium* spp. genome, different bio-evolution stages in the host and vector and antigenic variation for escaping from immune system, complicate the fight against malaria. Therefore, *Anopheles* spp. due to their behaviour and ability for malaria transmission, are one of the main targets for restricting this disease. Vaccines have been the primary objects to achieve this goal from previous years, but most of them have been designed based on parasite antigens [43]. Few studies have been performed to find the critical molecules in the vector, which are essential for sexual parasite development, and few vector-based antigens have been introduced as promising candidates for vaccine development against malaria [44]. Most of these molecules are considered as classical transmission-blocking vaccines, which are a category of VIMTs. According to the considerable results of AgAPN-1 as a TBV candidate for regions where *An. gambiae* is the main vector, AsAPN-1 was characterized in this study to provide the basic and necessary information for evaluating its potential role as a TBV candidate molecule in regions where *An. stephensi* plays the main role in malaria transmission.

According to the genomics results, the *apn*-*1* gene in *An. stephensi* has four introns and five exons. This structure is very similar to its counterpart gene in *An. gambiae* and the length of their coding sequences has high similarity. With regard to the structure of several reported isoforms of *apn* gene in different insects, it could be concluded that the characterized gene is the correct isoform of *agapn*-*1* in *An. stephensi*. Moreover, phylogenetic tree analysis of AsAPN-1and its comparison with some characterized APN proteins from other insects revealed that AsAPN-1 is located in the same branch with AgAPN-1. In addition, protein sequence alignment of the APN enzymes from the seven species of insects showed that the structural residues of their active sites are conserved, except the case that glutamic acid had been substituted with glutamine in AsAPN-1 at 186 position. Furthermore, involved residues in zinc binding motif are fully conserved. Another interesting point is peptide 9, a small protective peptide against *Plasmodium* spp. infection which has more than 50% similarity among the aligned sequences (Fig. 2). This fact is important for designing a universal VIMT to be applicable in different endemic malaria regions.

According to the predicted model and hydrophobicity analysis and acquired scores, it is predicted that AsAPN-1 would be a good antigen. In addition, it is interesting that peptide 9 is located in the surface of the protein and available for immune system recognition (Fig. 6a–c). These results and this level of accessibility are in accordance with the considered role for peptide 9 in sexual parasite development in the mosquito midgut.

Performed studies on the antigenicity of peptides according to the MHC presented type have shown that the longer peptides (10–15 residues) induce stronger CD4+ and CD8+ T-cell immunity responses in immunized hosts and are more efficient and stable than the shorter peptides [45]. For instance, Ekeruche-Makinde et al. [46] observed that the outcome of T-cell receptor/MHC interaction is dependent on the peptide length and the sequence identity of the MHC-bound peptide [46]. Immunoinformatics analysis showed that the length of the in silico predicted antigenic peptides are almost 9–14 residues and it is expected that the processing and presenting of AsAPN-1 on MHC-II groove be done well in antigen processing cells of immune system. Among the predicted antigenic peptides of AsAPN-1, there are only one fragments that their lengths are 27 residues and these results propose a good presentation of AsAPN-1 to immune system (Table 5). Therefore, peptides might not be able to bind the MHC groove in suitable and effective form. It is interesting that peptide 9, which was identified and confirmed by Dinglasan et al. as the shortest and best protective peptide in the experimental assays, acquired good scores for presenting with the common MHC-II alleles in Iran [21].

Genetic diversity of the MHC plays an central role in antigen recognition and strength of immune responses [47]. Each individual has specific alleles which may be different in distinct geographical regions [48]. Therefore, for designing an effective recombinant immunogenic vaccine, considering and evaluating the prevalence of an allele in the target area is crucial [49]. Draheim et al. study disclosed that the presentation of malaria antigen by MHC-II on dendritic cell is particularly important in induction of an effective immune response [50]. According to the *in silico* analysis, it is predicted that nine antigenic peptides have the best scores for perfect processing and presentation ability with the DQA10102-DQB10602 and DR B1 alleles and those could be considered as candidates for developing a recombinant subunit vaccine (Additional file 3) [51]. A study on Fula ethnic group (the largest ethnic groups in the Sahel and West Africa) showed that the presented antigens by DQA-DQB are associated with the higher levels of antibody production [51]. With regard to the geographical location and ethnic genome polymorphisms, prediction of the immune response before any laboratory analysis is critical. Herein, HLA-DRB1 allele which is common among the Caucasian and sub-Saharan African populations and has been evaluated in the Dinglasan et al. study, has the best predicted binding efficiency in comparison to the other examined MHC alleles in our analysis. Furthermore, analysis using pyDockWEB and NetMHCII revealed that AsAPN-1 is well processed and presented by the common MHC-II alleles in Iran and potentially could be a suitable candidate molecule for vaccine development. In addition, NetMHCII scores and statistical data revealed that AsAPN-1 is better processed and presented by DP, DQ and DR alleles in comparison with AgAPN-1. With regard to the presence of *An. stephensi* in Iran and neighbouring countries and the frequency of the mentioned alleles in this region, this seems that there is a natural adaptation between the target antigen and immune system.

For achieving the best functional and native structure of the recombinant protein, it is necessary to evaluate the occurrence of PTM such as glycosylation, especially in vaccine candidate molecules. These modifications are highly sequence specific and dependent to the arrangement of residues in the primary structure of proteins [20]. Results of the PTM prediction revealed that AsAPN-1 could be modified by glycosylation at three residues in V2, N148, and N589 positions. It is noticeable that two of them have been located in N-terminal of AsAPN-1 and it should be considered that peptide 9, which is the best and short protective epitope in the Dinglasan et al. study, has been located in N-terminal region. Therefore, suitable host must be selected for recombinant expression of the target protein according to the predicted PTMs.

Antigen stability is an important determining factor for potency and efficacy of a vaccine candidate molecule. Variation in antigenic epitopes of a protein would not lead to the production of effective neutralizing antibodies by immune system. Therefore, it is necessary to consider the structurally stable antigens in the process of vaccine design. *In silico* analysis of the present study revealed that our target protein has a stable and rigid structure, which is suitable for recombinant antigen production except the first and second residues which are located in the N- terminal region and have variable structure. It is predicted that there are three variable regions in the C-terminal region. Therefore, according to this point and the results of Dinglasan et al. it is reasonable to consider N-terminal part, including peptide 9, for developing an efficient vaccine. Additionally, this location is represented by the most common MHC-II alleles in the selected area in this study and possibly other neighbor countries in the Middle East region. In addition, several previous investigations evaluated the protection efficacy of different segments of AgAPN1 [52]. According to the findings of Dinglasan et al. residues at positions 758 and 821 are the ookinete attachment sites that their blocking with antibodies could be resulted in inhibition of the sexual *P. falciparum* development in the mosquito midgut [53]. Moreover, based on the performed peptide mapping by Armistead et al., it was found that the produced antibodies against the peptide 9 have the best inhibitory effects on ookinete attachment. In another study, Atkinson et al. performed peptide mapping which showed that the size of epitopes were 60 to 200 amino acids long. Their study revealed that some peptides, despite are on the surface, did not have the suitable performance, and only peptide 9, which has been located at residues 173-194, had the perfect inhibitory feature. According to our predictions, the 3D structure of these regions is identical to the reference molecule and those could be considered for an efficient regional VIMT development.

In addition, similar bioactivity of the recombinant AsAPN-1 and recombinant aminopeptidase enzyme of *Streptomyces griseus* (as standard) on specific substrate and the same inhibitory effect of 1,10-phenanthroline on enzymatic activity of them suggest that these two proteins are belonged to a same enzyme super-family. These data confirm the performed structural analysis on AsAPN-1 and show that AsAPN-1 is belong to the aminopeptidase superfamily.

## Conclusion

The importance of VIMTs and efficient ookinete-blocking activity of the antibodies produced against AgAPN-1, AsAPN-1 as its counterpart in *An. stephensi* was characterized in this study. Structural *in silico* analysis revealed that the critical residues in AsAPN-1 have very close similar topology with their counterparts in AgAPN-1, especially in peptide 9. Moreover, biological activity assay with specific substrate and inhibitor confirmed that the characterized protein (AsAPN-1) is related to the aminopeptidase superfamily and confirms the in silico structural analysis and docking results with specific inhibitor. These findings are hopeful for the future steps and pave the road for designing a new vector-based VIMT for countries that *An. stephensi* is the main malaria vector. However, there is a black box regarding the distance between the peptide-9 and ookinete attachment site in AgAPN-1 and AsAPN-1 and their relation in structure that needs to be answered in the future studies: how the produced polyclonal antibodies against the peptide-9 could inhibit sexual parasite development while the ookinete attachment site has been located in another and distinctive domain?

## Supplementary information

**Additional file 1.** Antigenic peptides of AsAPN-1.

**Additional file 2.** Antigenic peptides of AgAPN-1.

**Additional file 3.** Predicted peptides which are presented by MHC-II from AsAPN-1.

**Additional file 4.** Predicted peptides which are presented by MHC-II from AgAPN-1.

## Data Availability

The sequences which obtained and/or analysed during the current study were deposited in the GenBank database under the accession numbers (1985952) for 5ʹgenome walking, (1990583) for 3ʹRACE and (2017959) for full length of the *Asapn*-*1* gene. All the other related data are included in the article.

## Abbreviations

APN1: Aminopeptidase N-1
VIMT: Vaccines that interrupt malaria transmission
AgAPN-1: *An. gambiae*, aminopeptidase N-1
Sf9: Spodopterafrugiperda
Asapn-1: *An. stephensi* aminopeptidase N-1
CPBAg1: Carboxy peptidase B1
TBV: Transmission-blocking vaccine
PII: Pasteur Institute of Iran
MVRG: Malaria and Vector Research Group
PCR: Polymerase chain reaction
SVM: Support vector machines
Cɑ-RMSD: Alpha carbon root-mean-square deviation
MHC-II: Major histocompatibility complex II
4WZ9: AgAPN-1 PDB accession Number
PTMs: Post translation modifications
3D: Three-dimensional structure
QMEAN: Qualitative model energy analysis
GMQE: Global model quality estimation
SB: Strong binding
WB: Weak binding
