# Supplementary material for: Identification, molecular characterization and expression of aminopeptidase N-1 (APN-1) from Anopheles stephensi in SF9 cell line as a candidate molecule for developing a vaccine that interrupt malaria transmission
Source: Malar J. 2020 Feb 19;19:79. doi: 10.1186/s12936-020-03154-3 (PMC7029531; doi:10.1186/s12936-020-03154-3)
Supplement: Supplementary file 1 — Additional file 1. Antigenic peptides of AsAPN-1. [file 12936_2020_3154_MOESM1_ESM.docx]

Additional file 1:

Table S1 Antigenic peptides of AsAPN-1

http://imed.med.ucm.es/Tools/antigenic.pl

| Protein | N | Start position | Sequence | End position |
| --- | --- | --- | --- | --- |
|  | 1 | 4 | SKAVTVGCCAA | 14 |
|  | 2 | 35 | GSVGVAVNA | 43 |
|  | 3 | 45 | RKTSVHYD | 52 |
|  | 4 | 60 | SGSVAVTT | 67 |
|  | 5 | 70 | HTRGVSS | 76 |
|  | 6 | 83 | VTGAVGD | 89 |
|  | 7 | 114 | GYVSSYVA | 121 |
|  | 8 | 134 | ARMACYD | 140 |
|  | 9 | 142 | KATTVSTHST | 151 |
|  | 10 | 176 | SSYAVVSD | 183 |
|  | 11 | 188 | TRVYVRN | 194 |
| AsAPN-1 | 12 | 197 | TAAGVKKVDHGY | 208 |
|  | 13 | 227 | TYRDAVST | 234 |
|  | 14 | 242 | TTAHYAHW | 249 |
|  | 15 | 260 | ATYYAAHAYAYWN | 272 |
|  | 16 | 293 | SADRVAYKS | 301 |
|  | 17 | 324 | RAGAVDY | 330 |
|  | 18 | 337 | GVNGVTV | 343 |
|  | 19 | 378 | MYNYVHA | 384 |
|  | 20 | 397 | HYRSHACRSTRKHVGYYRV | 415 |
|  | 21 | 474 | NYHDVDAYGTDVVSN | 488 |
|  | 22 | 490 | SHKYVTTSWACSGYKD | 505 |
|  | 23 | 515 | GTGAVHDATVTYCY | 528 |
|  | 24 | 576 | RRRVVAVYSGSRVGVDAYMDAVVNVSS | 602 |
|  | 25 | 699 | TGSAAVT | 705 |
