# Supplementary material for: Identification, molecular characterization and expression of aminopeptidase N-1 (APN-1) from Anopheles stephensi in SF9 cell line as a candidate molecule for developing a vaccine that interrupt malaria transmission
Source: Malar J. 2020 Feb 19;19:79. doi: 10.1186/s12936-020-03154-3 (PMC7029531; doi:10.1186/s12936-020-03154-3)
Supplement: Supplementary file 2 — Additional file 2. Antigenic peptides of AgAPN-1. [file 12936_2020_3154_MOESM2_ESM.docx]

Additional file 2:

Table S2 Antigenic peptides of AgAPN-1

http://imed.med.ucm.es/Tools/antigenic.pl

| Protein | N | Start position | Sequence | End position |
| --- | --- | --- | --- | --- |
|  | 1 | 4 | VSGCCASA | 11 |
|  | 2 | 28 | ASDGVAAAVDR | 38 |
|  | 3 | 56 | GTVGVVATD | 64 |
|  | 4 | 72 | VMSSAKVSS | 80 |
|  | 5 | 114 | GYVSSYVA | 121 |
|  | 6 | 134 | ARMACYD | 140 |
|  | 7 | 143 | ATTVSTHSSY | 152 |
|  | 8 | 173 | STYAVVSD | 180 |
|  | 9 | 183 | GARVYVR | 189 |
|  | 10 | 194 | TAAGVKKVDD | 203 |
|  | 11 | 223 | VTYRANAVST | 232 |
|  | 12 | 239 | VATTAHYAHW | 248 |
|  | 13 | 259 | ATYYADM | 265 |
| **AgAPN-1** | 14 | 268 | GYWNVYA | 274 |
|  | 15 | 291 | SADRVAY | 297 |
|  | 16 | 323 | RAGAVDY | 329 |
|  | 17 | 336 | GVNGVTV | 342 |
|  | 18 | 377 | MYNYVHA | 383 |
|  | 19 | 399 | VANWVNKVGYYRV | 411 |
|  | 20 | 469 | AYHNVDA | 475 |
|  | 21 | 478 | STDAVSD | 484 |
|  | 22 | 486 | THKYVTSTWACSMG | 499 |
|  | 23 | 513 | GTGAVHDASVTYCYG | 527 |
|  | 24 | 571 | RRRVVAY | 577 |
|  | 25 | 588 | DAVNVST | 594 |
|  | 26 | 651 | ATTTVTA | 657 |
|  | 27 | 676 | VTTTVVT | 682 |
|  | 28 | 690 | TGSAAVST | 697 |
