# Supplementary material for: Identification, molecular characterization and expression of aminopeptidase N-1 (APN-1) from Anopheles stephensi in SF9 cell line as a candidate molecule for developing a vaccine that interrupt malaria transmission
Source: Malar J. 2020 Feb 19;19:79. doi: 10.1186/s12936-020-03154-3 (PMC7029531; doi:10.1186/s12936-020-03154-3)
Supplement: Supplementary file 3 — Additional file 3. Predicted peptides which are presented by MHC-II from AsAPN-1. [file 12936_2020_3154_MOESM3_ESM.docx]

Additional file 3

Predicted peptides which are presented by MHC-II from AsAPN-1

http://www.cbs.dtu.dk/services/NetMHCII-2.2/

| Pos | Allele | Peptide | Affinity(nM) | Bind Level^*^ |
| --- | --- | --- | --- | --- |
| 1 |  | SRVGVDAYMDAVVNV | 55.9 |  |
| 2 |  | GSRVGVDAYMDAVVN | 60.1 |  |
| 3 |  | RVGVDAYMDAVVNVS | 63.8 |  |
| 4 |  | VGVDAYMDAVVNVSS | 74.9 |  |
| 5 |  | SGSRVGVDAYMDAVV | 76.5 |  |
| 6 |  | GVDAYMDAVVNVSST | 146.2 |  |
| 7 |  | RKHVGYYRVNYDDRN | 170.8 |  |
| 8 |  | KHVGYYRVNYDDRNW | 176.9 |  |
| 9 |  | TRKHVGYYRVNYDDR | 181.9 |  |
| 10 |  | TTAHYAHWGDVTWWY | 182.4 |  |
| 11 |  | STRKHVGYYRVNYDD | 188.9 |  |
| 12 |  | TAHYAHWGDVTWWYW | 192.6 |  |
| 13 |  | AHYAHWGDVTWWYWN | 201.8 |  |
| 14 | HLA-DQA10101-DQB10501 | ATTAHYAHWGDVTWW | 206.6 | WB |
| 15 |  | HVGYYRVNYDDRNWT | 207.7 |  |
| 16 |  | VDAYMDAVVNVSSTS | 244.5 |  |
| 17 |  | YAHWGDVTWWYWNGA | 246.5 |  |
| 18 |  | VGYYRVNYDDRNWTN | 292.6 |  |
| 19 |  | HYAHWGDVTWWYWNG | 298.3 |  |
| 20 |  | NATTAHYAHWGDVTW | 320.9 |  |
| 21 |  | AHWGDVTWWYWNGAT | 365.9 |  |
| 22 |  | WGDVTWWYWNGATYY | 369.7 |  |
| 23 |  | AGAMNWGVTYRDAVS | 396.6 |  |
| 24 |  | HWGDVTWWYWNGATY | 411.3 |  |
| 25 |  | GDVTWWYWNGATYYA | 426.6 |  |
| 26 |  | GAMNWGVTYRDAVST | 445.7 |  |
| 27 |  | VNTNWMYNYVHAKAD | 451.3 |  |
| 28 |  | RSTRKHVGYYRVNYD | 474.9 |  |
| 29 |  | DVTWWYWNGATYYAA | 475.3 |  |

Additional file 3

predicted peptides which are presented by MHC-II from AsAPN-1 (continue)

http://www.cbs.dtu.dk/services/NetMHCII-2.2/

| Pos | Allele | Peptide | Affinity(nM) | Bind Level |
| --- | --- | --- | --- | --- |
| 1 |  | RYATTSTSARMACYD | 20.3 |  |
| 2 |  | YATTSTSARMACYDA | 20.7 |  |
| 3 |  | ATTSTSARMACYDAK | 24.3 |  |
| 4 |  | RRYATTSTSARMACY | 25.0 |  |
| 5 |  | TTSTSARMACYDAKA | 30.0 |  |
| 6 |  | WWYWNGATYYAAHAY | 32.0 |  |
| 7 |  | TWWYWNGATYYAAHA | 32.4 |  |
| 8 |  | GKGVNGVTVAMRTWT | 36.9 |  |
| 9 |  | VTWWYWNGATYYAAH | 38.6 |  |
| 10 |  | KGVNGVTVAMRTWTN | 39.8 |  |
| 11 | HLA-DQA10102-DQB10602 | YAYWNTVAAMVDGAS | 39.9 | SB |
| 12 |  | AYAYWNTVAAMVDGA | 40.0 |  |
| 13 |  | WYWNGATYYAAHAYA | 40.4 |  |
| 14 |  | AGKGVNGVTVAMRTW | 41.0 |  |
| 15 |  | AYWNTVAAMVDGAST | 42.5 |  |
| 16 |  | GVNGVTVAMRTWTNA | 44.2 |  |
| 17 |  | STYMTNAVDAAGAMN | 44.9 |  |
| 18 |  | YSTYMTNAVDAAGAM | 45.6 |  |
| 19 |  | YRTKTNATTAHYAHW | 47.9 |  |
| 20 |  | RTKTNATTAHYAHWG | 48.0 |  |
| 21 |  | RVYVRNAATAAGVKK | 48.2 |  |
| 22 |  | TYMTNAVDAAGAMNW | 48.4 |  |
| 23 | Allele | TSTSARMACYDAKAT | 51.2 |  |
| 24 |  | TRVYVRNAATAAGVK | 51.9 |  |
| 25 |  | HAYAYWNTVAAMVDG | 52.2 |  |
| 26 |  | YMTNAVDAAGAMNWG | 55.8 |  |
| 27 |  | TYRTKTNATTAHYAH | 56.2 |  |
| 28 |  | GYSTYMTNAVDAAGA | 56.4 |  |
| 29 |  | TKTNATTAHYAHWGD | 57.3 |  |
| 30 |  | SAGKGVNGVTVAMRT | 58.0 |  |
| 31 | HLA-DQA10102-DQB10602 | VAYKSGSVNMMRHVG | 59.1 | WB |
| 32 |  | YWNTVAAMVDGASTR | 59.5 |  |
| 33 |  | YWNGATYYAAHAYAY | 60.0 |  |
| 34 |  | AYKSGSVNMMRHVGD | 61.0 |  |
| 35 |  | VYVRNAATAAGVKKV | 61.5 |  |
| 36 |  | GRRYATTSTSARMAC | 63.2 |  |
| 37 |  | GTRVYVRNAATAAGV | 63.5 |  |
| 38 |  | VNGVTVAMRTWTNAG | 68.9 |  |
| 39 |  | MTNAVDAAGAMNWGV | 69.9 |  |
| 40 |  | RVAYKSGSVNMMRHV | 75.3 |  |
| 41 |  | RRRVVAVYSGSRVGV | 10.8 |  |
| 42 |  | RRVVAVYSGSRVGVD | 11.4 |  |
| 43 | HLA-DRB11501 | DRRRVVAVYSGSRVG | 14.0 | SB |
| 44 |  | ADRRRVVAVYSGSRV | 15.0 |  |
| 45 |  | RVVAVYSGSRVGVDA | 15.1 |  |
| Pos | Allele | Peptide | Affinity(nM) | Bind Level |
| 46 |  | VVAVYSGSRVGVDAY | 21.0 |  |
| 47 |  | ATYYAAHAYAYWNTV | 32.9 |  |
| 48 | HLA-DRB11501 | TYYAAHAYAYWNTVA | 37.1 | SB |
| 49 |  | YYAAHAYAYWNTVAA | 40.1 |  |
| 50 |  | VGVAVNAYRKTSVHY | 47.7 |  |
| 51 |  | YAAHAYAYWNTVAAM | 47.9 |  |
| 52 |  | GATYYAAHAYAYWNT | 48.7 |  |
| 53 |  | KVNTNWMYNYVHAKA | 54.6 |  |
| 54 |  | SVGVAVNAYRKTSVH | 54.7 |  |
| 55 |  | VNTNWMYNYVHAKAD | 57.1 |  |
| 56 | HLA-DRB11501 | VAVYSGSRVGVDAYM | 57.5 | WB |
| 57 |  | NGATYYAAHAYAYWN | 57.8 |  |
| 58 |  | NTNWMYNYVHAKADN | 61.2 |  |
| 59 |  | RTTVDKTMSSYAVVS | 63.5 |  |
| 60 |  | GSVGVAVNAYRKTSV | 63.5 |  |
| 61 |  | TRVYVRNAATAAGVK | 3.8 |  |
| 62 |  | RVYVRNAATAAGVKK | 4.2 |  |
| 63 |  | GTRVYVRNAATAAGV | 4.3 |  |
| 64 |  | VTYCYGMRTTGAAYY | 4.4 |  |
| 65 |  | VYVRNAATAAGVKKV | 4.9 |  |
| 66 |  | GATYYAAHAYAYWNT | 5.2 |  |
| 67 |  | TYCYGMRTTGAAYYY | 5.4 |  |
| 68 |  | DGTRVYVRNAATAAG | 5.6 |  |
| 69 |  | TVTYCYGMRTTGAAY | 5.6 |  |
| 70 |  | NGATYYAAHAYAYWN | 6.0 |  |
| 71 |  | YVRNAATAAGVKKVD | 6.2 |  |
| 72 |  | ATYYAAHAYAYWNTV | 6.4 |  |
| 73 |  | RDGTRVYVRNAATAA | 6.7 |  |
| 74 |  | WNGATYYAAHAYAYW | 6.8 |  |
| 75 | HLA-DRB10101 | AYAYWNTVAAMVDGA | 6.8 | SB |
| 76 |  | RGRYAWAAANTASYN | 7.1 |  |
| 77 |  | YCYGMRTTGAAYYYR | 8.0 |  |
| 78 |  | RMACYDAKATTVSTH | 8.0 |  |
| 79 |  | AYYYRKMMDSKNART | 8.2 |  |
| 80 |  | RMTYRGRYAWAAANT | 8.4 |  |
| 81 |  | HAYAYWNTVAAMVDG | 8.4 |  |
| 82 |  | TYYAAHAYAYWNTVA | 8.5 |  |
| 83 |  | YRGRYAWAAANTASY | 8.7 |  |
| 84 |  | AAHAYAYWNTVAAMV | 8.7 |  |
| 85 |  | AHAYAYWNTVAAMVD | 8.7 |  |
| 86 |  | ATVTYCYGMRTTGAA | 9.0 |  |
| 87 |  | RVAYKSGSVNMMRHV | 9.3 |  |
| 88 |  | YWNGATYYAAHAYAY | 9.4 |  |
| 89 |  | GRYAWAAANTASYNS | 9.6 |  |
| 90 |  | YAYWNTVAAMVDGAS | 10.4 |  |
| Pos | Allele | Peptide | Affinity(nM) | Bind Level |
| 91 |  | ATYYAAHAYAYWNTV | 139.7 |  |
| 92 |  | TYYAAHAYAYWNTVA | 171.3 |  |
| 93 |  | SNDSHKYVTTSWACS | 197.4 |  |
| 94 |  | YYAAHAYAYWNTVAA | 228.4 |  |
| 95 | HLA-DPA10103-DPB10401 | DSHKYVTTSWACSGY | 244.8 | WB |
| 96 |  | NDSHKYVTTSWACSG | 262.1 |  |
| 97 |  | SHKYVTTSWACSGYK | 273.5 |  |
| 98 |  | AAHAYAYWNTVAAMV | 292.8 |  |
| 99 |  | YAAHAYAYWNTVAAM | 318.1 |  |
| 100 |  | HKYVTTSWACSGYKD | 320.9 |  |

Bind Level*: SB=strong binding, WB= weak binding
