# Supplementary material for: Identification, molecular characterization and expression of aminopeptidase N-1 (APN-1) from Anopheles stephensi in SF9 cell line as a candidate molecule for developing a vaccine that interrupt malaria transmission
Source: Malar J. 2020 Feb 19;19:79. doi: 10.1186/s12936-020-03154-3 (PMC7029531; doi:10.1186/s12936-020-03154-3)
Supplement: Supplementary file 4 — Additional file 4. Predicted peptides which are presented by MHC-II from AgAPN-1. [file 12936_2020_3154_MOESM4_ESM.docx]

Additional file 4

Predicted peptides which are presented by MHC-II from AgAPN-1

http://www.cbs.dtu.dk/services/NetMHCII-2.2/

| Pos | Allele | Peptide | Affinity(nM) | Bind Level^*^ |
| --- | --- | --- | --- | --- |
| 1 |  | WWYWNGATYYADMAY | 54.7 |  |
| 2 |  | WYWNGATYYADMAYG | 62.9 |  |
| 3 |  | YWNGATYYADMAYGY | 64.1 |  |
| 4 |  | WNGATYYADMAYGYW | 83.2 |  |
| 5 |  | YADMAYGYWNVYAMG | 102.2 |  |
| 6 |  | ADMAYGYWNVYAMGD | 107.3 |  |
| 7 |  | YYADMAYGYWNVYAM | 109.4 |  |
| 8 |  | DMAYGYWNVYAMGDG | 123.4 |  |
| 9 |  | WGNVSWWYWNGATYY | 147.6 |  |
| 10 |  | NGATYYADMAYGYWN | 150.2 |  |
| 11 |  | MAYGYWNVYAMGDGA | 151.7 |  |
| 12 |  | HWGNVSWWYWNGATY | 167.8 |  |
| 13 |  | GNVSWWYWNGATYYA | 168.2 |  |
| 14 |  | NVSWWYWNGATYYAD | 186.6 |  |
| 15 | HLA-DQA10101-DQB10501 | AHWGNVSWWYWNGAT | 188.1 | WB |
| 16 |  | TWACSMGYTDCMKTA | 200.8 |  |
| 17 |  | STWACSMGYTDCMKT | 206.8 |  |
| 18 |  | ATYYADMAYGYWNVY | 206.9 |  |
| 19 |  | VNKVGYYRVNYDHNW | 216.0 |  |
| 20 |  | VSWWYWNGATYYADM | 248.1 |  |
| 21 |  | GATYYADMAYGYWNV | 251.6 |  |
| 22 |  | WACSMGYTDCMKTAA | 253.8 |  |
| 23 |  | NKVGYYRVNYDHNWT | 255.8 |  |
| 24 |  | TSTWACSMGYTDCMK | 263.9 |  |
| 25 |  | YAHWGNVSWWYWNGA | 263.9 |  |
| 26 |  | AYGYWNVYAMGDGAS | 277.4 |  |
| 27 |  | WVNKVGYYRVNYDHN | 283.1 |  |
| 28 |  | SWWYWNGATYYADMA | 293.7 |  |
| 29 |  | TYYADMAYGYWNVYA | 293.7 |  |
| 30 |  | KVGYYRVNYDHNWTN | 311.5 |  |

Bind Level*: SB=strong binding, WB= weak binding

Additional file 4

predicted peptides which are presented by MHC-II from AgAPN-1 (continue)

http://www.cbs.dtu.dk/services/NetMHCII-2.2/

| Pos | Allele | Peptide | Affinity(nM) | Bind Level |
| --- | --- | --- | --- | --- |
| 1 |  | RYATTSTSARMACYD | 20.3 |  |
| 2 |  | YATTSTSARMACYDG | 20.8 |  |
| 3 |  | GMSNAAATTTTAATT | 24.1 |  |
| 4 |  | ATTSTSARMACYDGK | 24.4 |  |
| 5 |  | RRYATTSTSARMACY | 25 |  |
| 6 |  | GGMSNAAATTTTAAT | 25.9 |  |
| 7 |  | MSNAAATTTTAATTT | 29.5 |  |
| 8 |  | TTSTSARMACYDGKA | 31.1 |  |
| 9 | HLA-DQA10102-DQB10602 | NGGMSNAAATTTTAA | 31.6 | SB |
| 10 |  | TTTTAATTTVTAGTT | 36.8 |  |
| 11 |  | GKGVNGVTVAMRTWT | 36.9 |  |
| 12 |  | ATTTTAATTTVTAGT | 38.9 |  |
| 13 |  | KGVNGVTVAMRTWTN | 39.8 |  |
| 14 |  | AGKGVNGVTVAMRTW | 41.0 |  |
| 15 |  | SNAAATTTTAATTTV | 43.8 |  |
| 16 |  | GVNGVTVAMRTWTNA | 44.2 |  |
| 17 |  | VNGGMSNAAATTTTA | 47.1 |  |

Additional file 4

predicted peptides which are presented by MHC-II from AgAPN-1 (continue)

http://www.cbs.dtu.dk/services/NetMHCII-2.2/

| Pos | Allele | Peptide | Affinity(nM) | Bind Level |
| --- | --- | --- | --- | --- |
| 1 |  | ASYYRKMMNSKNART | 4.3 |  |
| 2 |  | VTYCYGMRSASYYRK | 4.7 |  |
| 3 |  | SASYYRKMMNSKNAR | 4.8 |  |
| 4 |  | SYYRKMMNSKNARTM | 5.0 |  |
| 5 |  | TYCYGMRSASYYRKM | 5.4 |  |
| 6 |  | SVTYCYGMRSASYYR | 5.8 |  |
| 7 |  | RSASYYRKMMNSKNA | 6.0 |  |
| 8 |  | YYRKMMNSKNARTMD | 6.1 |  |
| 9 |  | ARVYVRNANATAAGV | 6.2 |  |
| 10 |  | RTGVDAMDAVNVSTS | 6.4 |  |
| 11 |  | RNRYAWTAANVATYN | 6.7 |  |
| 12 |  | YCYGMRSASYYRKMM | 6.8 |  |
| 13 |  | RVYVRNANATAAGVK | 7.2 |  |
| 14 |  | YRNRYAWTAANVATY | 7.8 |  |
| 15 | HLA-DRB10101 | GATYYADMAYGYWNV | 7.8 | SB |
| 16 |  | MRSASYYRKMMNSKN | 8.3 |  |
| 17 |  | ASVTYCYGMRSASYY | 8.6 |  |
| 18 |  | GRTGVDAMDAVNVST | 8.6 |  |
| 19 |  | YRKMMNSKNARTMDS | 9.1 |  |
| 20 |  | NRYAWTAANVATYNN | 9.4 |  |
| 21 |  | RMTYRNRYAWTAANV | 9.7 |  |
| 22 |  | TGVDAMDAVNVSTST | 10.1 |  |
| 23 |  | GVTYRANAVSTYRGK | 10.5 |  |
| 24 |  | VYVRNANATAAGVKK | 10.6 |  |
| 25 |  | GARVYVRNANATAAG | 10.7 |  |
| 26 |  | CYGMRSASYYRKMMN | 10.8 |  |
| 27 |  | ATYYADMAYGYWNVY | 11.1 |  |
| 28 |  | RYAWTAANVATYNNR | 11.6 |  |
| 29 |  | NGATYYADMAYGYWN | 11.7 |  |
| 30 |  | WGVTYRANAVSTYRG | 12.0 |  |

Additional file 4

predicted peptides which are presented by MHC-II from AgAPN-1 (continue)

http://www.cbs.dtu.dk/services/NetMHCII-2.2/

| Pos | Allele | Peptide | Affinity(nM) | Bind Level |
| --- | --- | --- | --- | --- |
| 1 |  | RRRVVAYSGGRTGVD | 20.0 |  |
| 2 |  | DRRRVVAYSGGRTGV | 20.9 |  |
| 3 |  | RRVVAYSGGRTGVDA | 26.5 |  |
| 4 |  | ADRRRVVAYSGGRTG | 32.0 | SB |
| 5 |  | RVVAYSGGRTGVDAM | 36.3 |  |
| 6 |  | RADRRRVVAYSGGRT | 37.4 |  |
| 7 |  | AHYAHWGNVSWWYWN | 46.7 |  |
| 8 |  | TAHYAHWGNVSWWYW | 50.4 |  |
| 9 |  | HYAHWGNVSWWYWNG | 51.2 |  |
| 10 | HLA-DRB11501 | KVNTNWMYNYVHAKA | 54.6 |  |
| 11 |  | TYCYGMRSASYYRKM | 55.2 |  |
| 12 |  | VNTNWMYNYVHAKAD | 57.1 |  |
| 13 |  | TTAHYAHWGNVSWWY | 57.5 | WB |
| 14 |  | YCYGMRSASYYRKMM | 59.0 |  |
| 15 |  | NTNWMYNYVHAKADN | 61.2 |  |
| 16 |  | VTYCYGMRSASYYRK | 63.9 |  |
| 17 |  | YAHWGNVSWWYWNGA | 70.6 |  |
| 18 |  | SVTYCYGMRSASYYR | 73.3 |  |
| 19 |  | TNWMYNYVHAKADND | 79.7 |  |
| 20 |  | CYGMRSASYYRKMMN | 79.8 |  |

Additional file 4

predicted peptides which are presented by MHC-II from AgAPN-1 (continue)

http://www.cbs.dtu.dk/services/NetMHCII-2.2/

| Pos | Allele | Peptide | Affinity(nM) | Bind Level^*^ |
| --- | --- | --- | --- | --- |
| 1 |  | DDTHKYVTSTWACSM | 207.9 |  |
| 2 |  | SDDTHKYVTSTWACS | 236.9 |  |
| 3 |  | DTHKYVTSTWACSMG | 284.3 |  |
| 4 |  | THKYVTSTWACSMGY | 322.6 |  |
| 5 | HLA-DPA10103-DPB10401 | YYADMAYGYWNVYAM | 336.3 | WB |
| 6 |  | ATYYADMAYGYWNVY | 341.5 |  |
| 7 |  | TYYADMAYGYWNVYA | 368.3 |  |
| 8 |  | YADMAYGYWNVYAMG | 373.1 |  |
| 9 |  | ADMAYGYWNVYAMGD | 414.2 |  |
| 10 |  | HKYVTSTWACSMGYT | 438.9 |  |

Bind Level*: SB=strong binding, WB= weak binding
